# Integrated High‐Throughput and Machine Learning Methods to Accelerate Discovery of Molten Salt Corrosion‐Resistant Alloys

**DOI:** 10.1002/advs.202200370

**Published:** 2022-05-07

**Authors:** Yafei Wang, Bonita Goh, Phalgun Nelaturu, Thien Duong, Najlaa Hassan, Raphaelle David, Michael Moorehead, Santanu Chaudhuri, Adam Creuziger, Jason Hattrick‐Simpers, Dan J. Thoma, Kumar Sridharan, Adrien Couet

**Affiliations:** ^1^ Department of Engineering Physics University of Wisconsin Madison WI 53706 USA; ^2^ Applied Materials Division Argonne National Laboratory Lemont IL 60607 USA; ^3^ National Institute of Standard and Technology Gaithersburg MD 20899 USA; ^4^ Department of Materials Science and Engineering University of Wisconsin Madison WI 53706 USA

**Keywords:** additive manufacturing, corrosion, high‐throughput methods, machine learning, molten salt

## Abstract

Insufficient availability of molten salt corrosion‐resistant alloys severely limits the fruition of a variety of promising molten salt technologies that could otherwise have significant societal impacts. To accelerate alloy development for molten salt applications and develop fundamental understanding of corrosion in these environments, here an integrated approach is presented using a set of high‐throughput (HTP) alloy synthesis, corrosion testing, and modeling coupled with automated characterization and machine learning. By using this approach, a broad range of Cr—Fe—Mn—Ni alloys are evaluated for their corrosion resistances in molten salt simultaneously demonstrating that corrosion‐resistant alloy development can be accelerated by 2 to 3 orders of magnitude. Based on the obtained results, a sacrificial protection mechanism is unveiled in the corrosion of Cr—Fe—Mn—Ni alloys in molten salts which can be applied to protect the less unstable elements in the alloy from being depleted, and provided new insights on the design of high‐temperature molten salt corrosion‐resistant alloys.

## Graphical abstract

An integrated approach using a set of high‐throughput alloy synthesis, corrosion testing, and modeling coupled with automated characterization and machine learning is developed to accelerate the molten salt corrosion‐resistant alloy development for molten salt applications by 2 to 3 orders of magnitude.

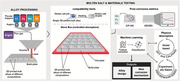

## 1 Introduction

Molten salts have many attractive properties including: i) relatively low melting point and high boiling point, ii) high thermal conductivity, iii) large heat capacity, and iv) excellent compositional stability. These properties make them leading candidates for high‐temperature applications such as coolant and fuel‐solvent for nuclear reactors,^[^ ^1^ ^]^ extraction media for pyroprocessing of spent nuclear fuel,^[^ ^2^ ^]^ thermal energy storage and heat transfer fluid of concentrated solar power,^[^ ^3^ ^]^ and battery electrolytes.^[^ ^4^ ^]^ One major concern in the deployment of these applications is material's corrosion in molten salts. Indeed, the protective oxide layer relied upon for the corrosion resistance in most aqueous solutions or oxidative environments is rendered unstable in high‐temperature molten salts, especially molten chloride and fluoride salts.^[^ ^5^ ^]^ Because of the strong electronegativity of chlorine and fluorine anions, these molten salts typically tend to destabilize the protective oxide layers, resulting in dissolution of the least thermodynamically stable element from the alloy into the salt. For example, Cr, one of the main constituents for all high‐temperature (>700 °C) American Society of Mechanical Engineers (ASME) code certified alloys, forms relatively stable metal halides and is particularly susceptible to dissolution in salts, such that all these alloys experience unacceptable corrosion rates in molten salts. Therefore, identifying corrosion resistant alloys compatible with high‐temperature molten salt environments is crucial for the large‐scale deployment of molten salt technologies.

Currently, the most promising alloy for high‐temperature molten salt applications is Hastelloy‐N, a Ni‐based alloy, containing 16Mo—7Cr—5Fe and other minor alloying elements.^[^ ^6^ ^]^ However, this alloy has limited high‐temperature creep resistance and its down‐selection was based on the testing of nine alloys from the INOR series with varying Mo, Cr, Fe, Ti, Al, Nb, and W contents.^[^ ^7^ ^]^ One can legitimately question if no other alloys within this alloy system, or within other alloy systems, could exhibit higher corrosion resistance. However, current state‐of‐the‐art alloy processing and testing in molten salt environments are not compatible with such an exploratory search and the immediate needs for molten salt technology deployment. For instance, arc melting,^[^ ^8^ ^]^ field assisted sintering or casting^[^ ^9^ ^]^ can only melt one ingot composition at a time, and the manufactured materials have to be annealed, cut, and further prepared for testing. In addition, only one alloy can be tested at a time for its corrosion behavior in a given test cell, and moreover these corrosion experiments usually have to be performed continuously for up to a few thousand hours.^[^ ^10^, ^11^ ^]^ This paradigm results in an extremely lengthy processing/testing/characterization pathway if one wishes to explore a large compositional map for an alloy system. Consequently, the material development processes and screening for high‐temperature molten salt technologies have lagged behind.

**Figure 1:**
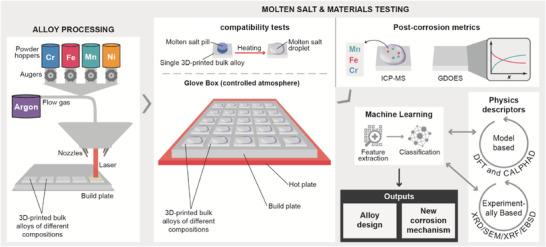
Schematic of the developed HTP and automated methods for corrosion‐resistant alloy development.

To accelerate the search for corrosion resistant alloys in molten salt and better understand the corrosion mechanism in molten salt across a wide range of conditions, HTP experimental and modeling techniques have been developed and integrated in the present study as presented in **Figure** **1**. First, an HTP alloy synthesis technique, namely in situ alloying using additive manufacturing, was employed to print bulk alloys of different selected compositions. The laser engineered net shaping (LENS) process was adopted,^[^ ^12^ ^]^ such that any alloy composition can be manufactured in a few minutes. HTP corrosion tests were also designed by melting molten salt pills on top of each printed bulk alloy at 500 °C. The corrosion behavior of each printed alloy was assessed by a series of automated material characterization techniques such as glow–discharge optical emission spectroscopy (GDOES) and inductively coupled plasma mass spectrometry (ICP‐MS). In addition, HTP first‐principle density functional theory (DFT) calculations of surface energy and work function were coupled with a calculation of phase diagram (CALPHAD) method to rank the corrosion resistance of different printed alloys. Finally, features extraction and cross‐validation were performed by machine learning (ML) based methods to determine the dependencies of corrosion resistance on alloys’ physical parameters. This HTP platform has been experimented for the first time to exploit the correlation between the Mn and Fe dissolutions in Cr–Fe–Mn–Ni alloys and the alloys’ corrosion resistance. A unique high temperature sacrificial corrosion mechanism in Cr–Fe–Mn–Ni alloys exposed to molten salt has been unveiled and discussed. The present study paves the way for accelerating the discovery of corrosion resistant alloys in molten salts, and serves as an example of alloy design for extreme environments using HTP and/or automated methods coupled to data analytics.

## 2 Results

### 2.1 Additive Manufacturing of Cr—Fe—Mn—Ni Alloys

**Figure 2:**
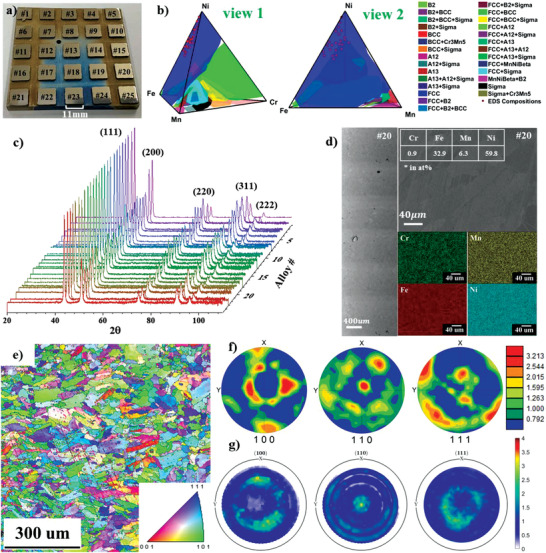
Characterizations of precorroded Cr—Fe—Mn—Ni alloys. a) The 25 printed Cr—Fe—Mn—Ni alloys with different compositions after homogenizing, aging, and polishing. b) Compositions of the printed 25 Cr—Fe—Mn—Ni alloys identified by EDS with the CALPHAD equilibrium phases of Cr—Fe—Mn—Ni alloy system. c) XRD patterns of the 25 printed alloys after heat treatments. d) SEM and EDS analysis of alloy #20 (note that signal to noise ratio in the Cr map is low). e) EBSD IPF map of the surface normal to the build direction of alloy #21 (0.9 at% Cr, 30.5 at% Fe, 14.7 at% Mn, and 53.9 at% Ni). f) EBSD pole figures corresponding to the area shown in (e). g) XRD pole figures of alloy #21.

Cr—Fe—Mn—Ni alloy systems have a relatively well‐known set of properties inherited from the stainless‐steel family and thus represents a model alloy system to serve as a case study in the HTP platform investigation shown in Figure 1. Additive manufacturing was selected to perform in situ alloying through the LENS process, as developed in ref. [13]. By using this technology, 25 bulk Cr—Fe—Mn—Ni alloys with dimensions of 11 mm × 11 mm × 2 mm were printed on a 316 stainless steel build plate (**Figure** **2**a). The alloy compositions were targeted toward high Ni and low Cr to stabilize a single‐phase FCC structure across all compositions and follow molten salt alloy design baseline requirements. To remove the dendritic compositional segregation and residual stresses, which are often observed in the as‐printed alloys,^[^ ^14^ ^]^ the 25 printed alloys were simultaneously homogenized at 1000 °C for 24 h in a vacuum furnace with a base pressure of 10^–6^ torr. A follow‐up heat‐treatment was also performed at 700 °C for 24 h to obtain a stable microstructure of the printed alloys for subsequent corrosion testing. After heat treatment, the printed alloys were annealed, leveled, polished, and labeled as indicated in Figure 2a. The compositions of the printed alloys were measured by automated energy dispersive spectroscopy (EDS) and the results are plotted in Figure 2b. X‐ray fluorescence (XRF) was also used to accelerate chemical characterization of the alloys and the results match the compositions obtained by local EDS within a few at% (see Supporting Information). As expected from the targeted compositions, the final compositions are Ni‐rich with very low Cr content. The detailed information regarding the composition of each printed alloy can be found in Table S1, Supporting Information. The equilibrium phases of the Cr—Fe—Mn—Ni system at 700 °C were simulated using CALPHAD modeling based on the PanHEA database of the Pandat software (version 2020, https://computherm.com/) and the results are shown in Figure 2b, predicting that all the printed alloys are single‐phase FCC.

Automated X‐ray diffraction (XRD) was performed on each printed alloy after heat treatment and the profiles are presented in Figure 2c. The results show that all alloys are indeed single‐phase FCC, which is consistent with the CALPHAD predictions. The 2*θ* peak position varies slightly between each sample, which is likely due to the change in lattice parameter brought by different compositions (see Table S2, Supporting Information). The microstructures of all the printed alloys were examined by automated scanning electron microscopy (SEM) before corrosion except for alloy #1, which showed an irregular shape and severe surface defects resulting from the printing process and was discarded from the study. Figure 2d illustrates an example of SEM/EDS on a printed alloy surface: alloy #20 from which no defects of cracking or craters were found on the polished alloy surfaces. Spherical porosity and defects from inadequate fusion were sporadically observed as is typical in additive manufacturing technology.^[^ ^15^ ^]^ These pores and defects could be due to the unmelted powder particles during the additive manufacturing process. Higher magnification SEM micrographs showed limited evidence of microscale printing defects. Elemental EDS mapping also revealed that the printed alloys are compositionally homogeneous. These automated characterization methods demonstrate that the LENS in situ alloying is an ideal HTP method to manufacture bulk alloys for corrosion experiments. Additional SEM images and EDS mapping for other printed alloys are shown in Figures S1–S3, Supporting Information. It is worth mentioning that all heat treatments and characterizations were performed while the samples were still attached to the build plate, allowing for automation of the different processes. Electron backscatter diffraction (EBSD) was also performed on the top surface of multiple printed alloys to characterize grain morphology. As a typical example, Figure 2e,f display the EBSD scan results of alloy #21 (0.9 at% Cr, 30.5 at% Fe, 14.7 at% Mn, and 53.9 at% Ni). Figure 2e shows the inverse pole figure (IPF) map displaying the overall grain morphology and orientation at the surface. A distribution of small equiaxed grains and larger columnar grains was observed. These columnar grains are irregularly shaped and a majority of them have a high aspect ratio. The microstructure of the alloy exhibits a strong (110) texture as evidenced by the EBSD pole figures in Figure 2f. To increase the throughput of the sample texture information, XRD pole figures were also performed on the same set of alloys. The XRD pole figure results on alloy #21 are shown in Figure 2g which matches the EBSD results quite well, showing the relatively high (110) surface texture at the center. This means that (110) is prevalently parallel to the alloy surface. Based on the similar microstructures identified by the material characterization (EBSD and XRD pole figure) of the five representative printed alloys as shown in Figures S4 and S5, Supporting Information, all printed alloys are expected to exhibit similar grain morphologies, grain size distributions, and texture, resulting from the same in situ alloying parameters used in the LENS manufacturing process.

### 2.2 HTP Materials/Molten Salt Compatibility Test

**Figure 3:**
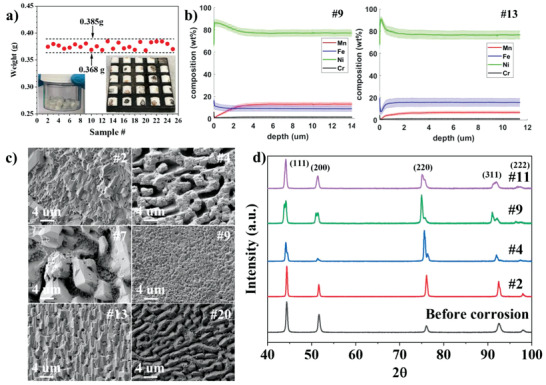
HTP corrosion test of the printed alloys in LiCl‐KCl‐2 wt% EuCl3 molten salt at 500 °C. a) Salt pills (inserted picture at left bottom corner) and their mass for corrosion test on each corresponding printed alloy, and the salt droplets solidified on each of the additive manufactured alloys after corrosion experiment (inserted picture at right bottom corner). b) GDOES analyses of two representative printed alloys after corrosion test, alloy #9 and alloy #13 (the shade area represents the uncertainty of the measured value). c) SEM micrographs of the top surfaces of a few representative printed alloys after corrosion test: alloy #2, alloy #4, alloy #7, alloy #9, alloy #13, and alloy #20. d) XRD patterns on the top surfaces of a few representative printed alloys after corrosion test: alloy #2, alloy #4, alloy #9, and alloy #11, the XRD pattern before corrosion used as the reference is taken from alloy #2.

HTP molten salt corrosion experiments were carried out on each of the printed alloys, while still attached to the build plate, to study their corrosion resistance in molten salt. LiCl‐KCl eutectic salt (44 wt% LiCl‐56 wt% KCl, melting point: 353 °C), the extraction medium of pyroprocessing of spent nuclear fuel, was used for the corrosion experiment. 2 wt% EuCl_3_ salt was added into LiCl‐KCl eutectic salt to promote the overall corrosion driving force by increasing the redox potential of the salt,^[^ ^16^ ^]^ such that the corrosion effects could be observed over a relatively short exposure time. To perform the corrosion test, salt pills consisting of LiCl‐KCl‐2 wt% EuCl_3_ were prepared (**Figure** **3**a) by a standard salt pelletizing procedure. The mass of each salt pill prepared for the corrosion test is illustrated in Figure 3a which shows that the pill's mass was controlled in the range of 0.368 to 0.385 g. Due to the relatively small variations in salt pill mass, the volume of salt to alloy surface area ratio is considered constant for all the tested alloys. The solid salt pills were placed on the polished surface of each printed alloy and melted into droplets at the temperature of 500 °C by a heating plate as described in the compatibility test of Figure 1.The corrosion test was performed for 96 h inside an inert atmospheric glovebox (O_2_<2 ppm, H_2_O<0.1 ppm). After the corrosion test, the melted molten salt droplets cooled down to room temperature, solidified as shown in Figure 3a, and were extracted. It is worth noting that although the droplet contact angle was not measured during the experiment, the molten droplets remained on the printed alloys and no spillage was observed at all, as evidenced in Figure 3a.

Each printed alloy was cleaned ultrasonically after corrosion test and a series of automated material characterization analyses were performed to characterize the corrosion performance. First, SEM was used to evaluate the corrosion attack on the surface of each printed alloy. The SEM micrographs of a few representative printed alloys are shown in Figure 3c. Differences in surface corrosion attack of the various alloys is evident. Detailed information of the surface corrosion attacks of other printed alloys are presented in Figures S6 and S7, Supporting Information. Automated XRD was used to identify any possible phase changes in the near surface regions of the printed alloys during the corrosion test. In Figure 3d, small peak splitting was observed on the XRD patterns of the post‐corrosion alloys. A similar phenomenon also appeared on the post‐corrosion Ni‐201 alloy as reported in a previous study.^[^ ^17^ ^]^ However, in the present study, the extent and 2*θ* location of peak splitting were found to vary among the different printed alloys (see XRD patterns of all other post‐corrosion printed alloys in Figure S8, Supporting Information). The peak splitting is likely a result of the change in lattice parameter at the near surface region, induced by the changes in composition of the FCC phase (depletion of elements, and injection of vacancies). Overall, no significant phase change was observed, confirming that the alloys remained single‐phase FCC during the corrosion test.

**Figure 4:**
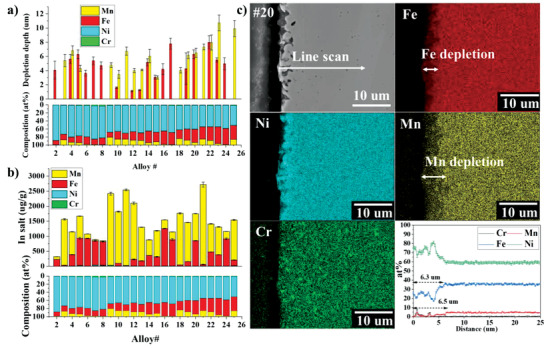
Corrosion resistance identification. a) Corrosion attack depth of different printed alloys. b) Concentrations of dissolved ions in molten salt during the corrosion testing for different printed alloys measured by ICP‐MS. c) SEM, EDS compositional mapping, and EDS line scan of the near surface region of alloy #20 (0.9 at% Cr, 32.9 at% Fe, 6.3 at% Mn, and 59.8 at% Ni).

To compare corrosion resistance, the corrosion attack depth was measured by automated GDOES analysis technique. Figure 3b gives an example of the obtained compositional profiles of Cr, Fe, Mn, and Ni as a function of depth (0 being the salt/sample interface) for the post‐corrosion alloy #9 (1.3 at% Cr, 12.6 at% Fe, 19.3 at% Mn, and 66.7 at% Ni) and alloy #13 (0.7 at% Cr, 19.2 at% Fe, 11.4 at% Mn, and 68.7 at% Ni) by GDOES (the GDOES profiles for other alloys are presented in Figure S9, Supporting Information). The elemental variations as a function of depth observed from the GDOES profile result from the elemental dissolution during corrosion. Based on Figure 3b, Mn is significantly depleted after the corrosion of alloy #9 while Mn and Fe are both depleted for alloy #13. Similarly, the depleted elements for the other printed alloys were found to be Mn or/and Fe as shown in Supporting Information. The depth corresponding to the variation of the depleted element usually signifies the “corrosion attack depth” or “depletion depth.” To systematically report the corrosion attack depth for each of the post‐corrosion printed alloys, an algorithm was developed to analyze the data measured by GDOES (see Experimental Section). The depletion depth of a given element for the different printed alloys are automatically generated and the results are shown in **Figure** **4**a.

ICP‐MS compositional analysis was also performed on the entire solidified salt droplet extracted from each printed alloy surface after corrosion test. The measured concentrations of the dissolved ions in molten salt are presented in Figure 4b, showing that Ni and Cr barely dissolved into the salt. This is expected since Ni has a higher standard redox potential for its metal chloride formation, and the printed alloys contain very little Cr. On the other hand, significant Fe and/or Mn concentrations were observed in the post‐corrosion salts. Due to the small volume of salt utilized in HTP corrosion experiment, saturations of dissolved Fe and Mn in salt droplet could interfere with the corrosion resistance identification of different printed alloys. To the authors’ knowledge, there is no solubility data of MnCl_2_ and FeCl_2_ in LiCl‐KCl molten salt reported in previous literatures. However, a few studies^[^ ^18^, ^19^ ^]^ performed electrochemical test of FeCl_2_ in LiCl‐KCl molten salt at 500 °C to study the diffusion coefficient of Fe^2+^ at different concentrations up to 5000 ppm and no salt saturation was observed. Electrochemical study of MnCl_2_ in LiCl‐KCl molten salt at 500 °C was conducted by Simpson et al.^[^ ^20^ ^]^ at different concentrations up to 8100 ppm and no salt saturation was observed as well. The concentrations of Fe^2+^ and Mn^2+^ in LiCl‐KCl molten salt reported in these studies^[^ ^18^, ^19^, ^20^ ^]^ are much higher than the total concentrations of corrosion products obtained by ICP‐MS (Figure 4b) in this study. Based on these literature inputs, it is unlikely that salt saturation occurred in the HTP corrosion experiment.

Figure 4b shows that alloy #2 has the lowest amount of corrosion products dissolved in the salt while it is the highest for alloy #21. These results are not exactly consistent with the depletion depth comparison obtained by GDOES. However, it should be noted that, as expected, the two performance metrics, depletion depth and overall concentration of the dissolved ions, do not necessarily follow the same alloy ranking order of corrosion resistance. This is because the two metrics are relatively different. An element that is over‐represented in the alloy composition would potentially lead to high concentration of dissolved ion upon corrosion even if its depletion depth is small. On the contrary, an alloying element with low concentration in the alloy but highly susceptible to local corrosion, such as grain boundary corrosion attack, would potentially have a relatively low concentration of dissolved ion but significant depletion depth. The concentration of the dissolved corrosion products can be used to evaluate the overall corrosion rate of one alloy in molten salt while the depletion depth can help understand the uniform and local corrosion attack occurring on the alloy. Some localized corrosions such as grain boundary corrosion attack or pitting might not be represented when using GDOES since the depletion depth obtained by this technique is based on the average composition variations in the sputtered crater area it creates. However, no deep localized corrosion was observed by SEM analysis of relatively large cross‐section area (see Figure S12, Supporting Information), such that GDOES should give a relatively good metric of elemental depletion depths.

To study the cross‐sectional microstructure and microchemistry of the post‐corrosion printed alloys, four alloys (#5, #7, #17, and #20) were cut through the center for SEM/EDS line scans and compositional maps. Figure 4c shows the SEM image, EDS compositional maps, and EDS line scan of alloy #20 (0.9 at% Cr, 32.9 at% Fe, 6.3 at% Mn, and 59.8 at% Ni) (similar results for the other three alloys are presented in Figure S10, Supporting Information). In Figure 4c, significant subsurface voids are observed. EDS mapping shows significant Fe and Mn depletions in the near surface region. EDS line scans were also performed and the corrosion attack depths were found to be about 6.3 µm for Fe and 6.5 µm for Mn. This data is included in Figure 4a together with the other results obtained by GDOES for the corrosion resistance comparison of different printed alloys.

### 2.3 Thermodynamic/Kinetic Modeling

**Figure 5:**
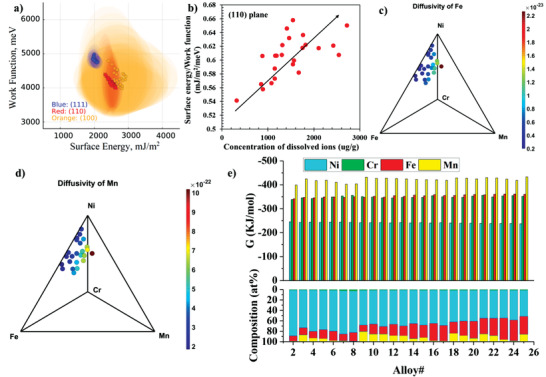
Thermodynamic and kinetic simulation. a) Surface energy and work function of planes (111), (110), and (100) of the printed alloys (shadowed eclipses represent 95% confidence bands/uncertainties). b) The correlation between the ratio of surface energy to work function and the overall concentrations of all the dissolved ions in post‐corrosion salt (arrow line is added to guide the eye). c) Tracer diffusivities of Fe in different printed alloys (in the unit of m^2^ s^−1^). d) Tracer diffusivities of Mn in different printed alloys (in the unit of m^2^ s^−1^). e) Gibbs free energy of CrCl_2_/Cr, FeCl_2_/Fe, MnCl_2_/Mn, and NiCl_2_/Ni for different printed alloys.

Surface energy indicates the stability of the surface of a material and can be used to infer how susceptible the material is against a thermodynamic reaction or chemical attack, while work function represents the difficulty of moving an electron from the surface of a material to vacuum above the surface. Thermodynamically, work function and surface energy are shown to be proportional and inversely proportional to the stability of a material in an oxidizing medium, respectively.^[^ ^21^, ^22^ ^]^ Therefore, they could represent good indicators to rank the inherent interface stability of the printed alloys. In the present study, first‐principle DFT combined with CALPHAD was used to calculate the surface energy and work function of the three common planes (111), (110), and (100) of the FCC structure of all the printed alloys and the results are shown in **Figure** **5**a. As can be seen from this figure, (111) appears to have lower surface energy and higher work function compared with (110) and (100) planes. This means that (111) is the most stable interface among the three planes. The ratio of surface energy to work function of the (110) plane, the most prevalent plane paralleling to the sample surface according to the pole figures in Figure 2f,g, is defined here to compare the corrosion resistance of different printed alloys based on the interface stability principle. Figure 5b shows an increasing trend between the ratio of surface energy to work function and the overall concentrations of the dissolved ions in post‐corrosion salt, suggesting that the alloy corrosion resistance could be related to the interface stability.

Other parameters expected to influence the corrosion resistance of elements in molten salts were also derived in this study. Based on the findings in the corrosion experiment study, Mn and Fe are the main depleted elements of the printed alloys during the corrosion. The Mn and Fe dissolution rates are very likely dependent on their diffusivities within the alloy matrix. Hence, the tracer diffusivities of Mn and Fe in the different printed alloys were calculated by the kinetics module in Pandat software (https://computherm.com/) using a mean field extrapolation approach on the databases reported in a previous study.^[^ ^23^ ^]^ Figure 5c,d show that the calculated tracer diffusivity of Mn is higher than that of Fe by about two orders of magnitude across all the printed alloys. Note that even if this method does not consider a specific diffusion mechanism, it is still consistent with the study^[^ ^24^ ^]^ in which Fe is reported to have a larger energy barrier for vacancy migration compared to Mn in equimolar Cr—Fe—Mn—Ni alloy. The Gibbs free energy of metal chloride formation (CrCl_2_/Cr, FeCl_2_/Fe, MnCl_2_/Mn, and NiCl_2_/Ni) were also calculated for different printed alloys using Equation (1) as done in ref. [25]

(1) $$G_{\mathrm{MC}l_{2}/M}=G_{\mathrm{MC}l_{2}/M}^{o}\;+RT\ln\left(\frac{\alpha_{\mathrm{MC}l_{2}}}{\alpha_{M}}\right)$$

where *M* represents Cr, Fe, Mn, and Ni, MCl_2_ is the metal chloride formed in molten salt during the corrosion, $G_{\mathrm{MC}l_{2}/M}^{o}$ is the standard Gibbs free energy of formation, which can be compiled from thermodynamic database, such as HSC Chemistry 6.0 (https://www.hsc‐chemistry.com/), *R* is the gas constant, *T* is the temperature (773 K in this study), $\alpha_{\mathrm{MC}l_{2}}$ is the activity of metal chloride in molten salt which is assumed to be 10^–6^,^[^ ^25^ ^]^ and *α*_M_ is the activity of M in the printed alloy calculated using the PanHEA database of Pandat software (version 2020, https://computherm.com/). As can be seen from Figure 5e, Mn has the lowest Gibbs free energy followed by Fe, Cr, and Ni. This means Mn is the element most thermodynamically reactive to form a dissolved metal chloride while Ni is the least reactive element.

## 3 Discussion

**Figure 6:**
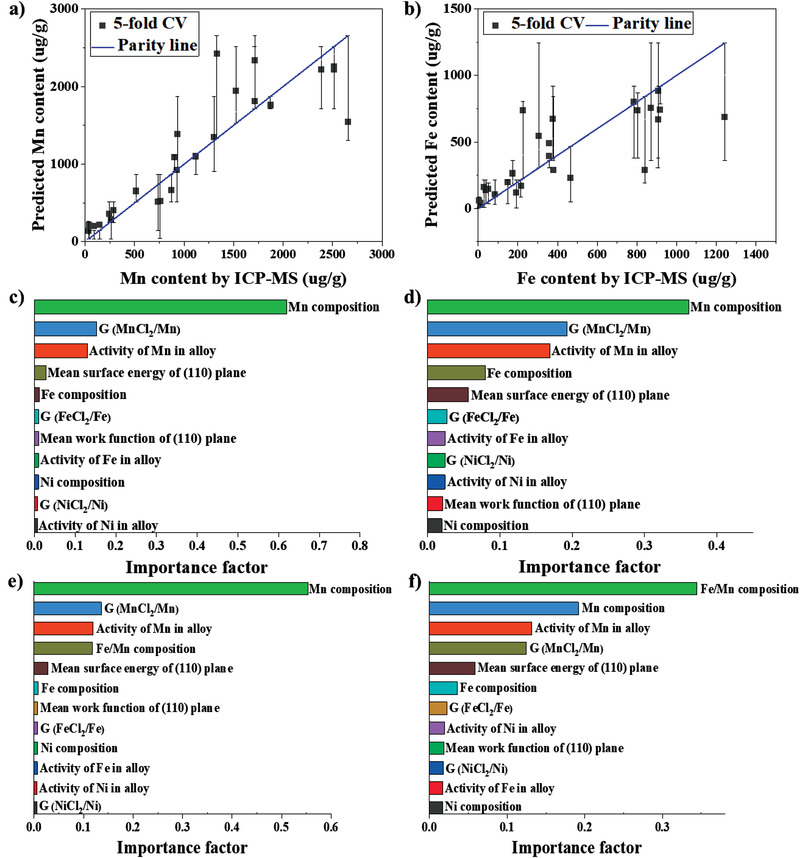
ML for corrosion resistance prediction. Predicted versus experimental values of a) Mn and b) Fe concentrations in post‐corrosion salt (error bar represents the 95% confidence interval of the prediction values). Input feature relative importance ranking order for contributing to the corrosions of c) Mn and d) Fe in molten salt without adding input feature of Fe/Mn composition ratio. Input feature relative importance ranking order for contributing to the corrosions of e) Mn and f) Fe in molten salt with the added input feature of Fe/Mn composition ratio.

This study includes the exposure of 24 different single‐phase FCC alloys from the Cr—Fe—Mn—Ni system to chloride salts at 500 °C for 96 h. The results mostly show Fe and/or Mn dissolutions, and four parameters are used to define the alloy corrosion resistance, namely the Fe and Mn depletion depths and the Fe and Mn dissolved concentrations in the salt. Alloy #2, which has the highest Ni content, shows the best corrosion resistance. This is expected since Ni is the most noble element in the alloy system of interest. However, ranking other alloys’ corrosion resistances based on their compositions or physical properties is more difficult. To assess the physical parameters affecting the corrosion resistance of the printed alloys, the dissolved Fe and Mn ion concentrations in the salt droplet after corrosion experiment were selected for ML data analysis based on the developed random Forest regression (RFR) model.^[^ ^26^ ^]^ This is because the localized corrosion attack, if any, might not be represented in the deletion depth obtained by GDOES. The objective of the ML‐based approach is to search for the most influential physical parameters in determining the concentrations of the dissolved ions in the post‐corrosion salt. In the developed model, the physical parameters selected as input features to train the active learning model include: i) element composition, ii) activity in the single FCC phase, iii) metal chloride Gibbs free energy of formation, iv) self‐diffusivity in the alloy, v) (110) plane surface energy, and vi) (110) plane work function. **Figure** **6**a,b show the RFR fivefold cross‐validation (CV) predicted concentrations of Mn and Fe in post‐corrosion salt with respect to the measured values by ICP‐MS from which a high consistence can be observed. The high correlation between CV predictions of dissolved Fe and Mn in post‐corrosion salt and the experimental values measured by ICP‐MS (Pearson's *R* value^[^ ^27^ ^]^ is 0.797 for Fe and 0.891 for Mn, respectively), which is on par with other published studies^[^ ^28^, ^29^ ^]^ on the implementation of ML to predict material properties from physical features, demonstrates the robustness of the RFR model and the reliability on the extraction of the importance rankings of input features. The extracted ranking of the input features for contributing to the depletions of Mn and Fe by importance scores are shown in Figure 6c,d. It was found that the physical parameters of Mn in the system, such as the composition of Mn in the alloy and Gibbs free energy of MnCl_2_/Mn, consistently ranks at the top in the importance score ranking for the predictions of Mn and Fe dissolved in the salt. This indicates that the behavior of Mn in the alloy plays a crucial role on the dissolutions of both Mn and Fe from the alloy into the molten salt. The effect of Mn related features on Fe dissolution indicates a possible sacrificial mechanism of Mn to protect Fe. Based on this finding, the Fe/Mn composition ratio was added as an input feature into the RFR model to study its potential influence on the depletion of Fe. The extracted input feature ranking in Figure 6e,f shows Fe/Mn composition ratio contributes the greatest to the depletion of Fe.

**Figure 7:**
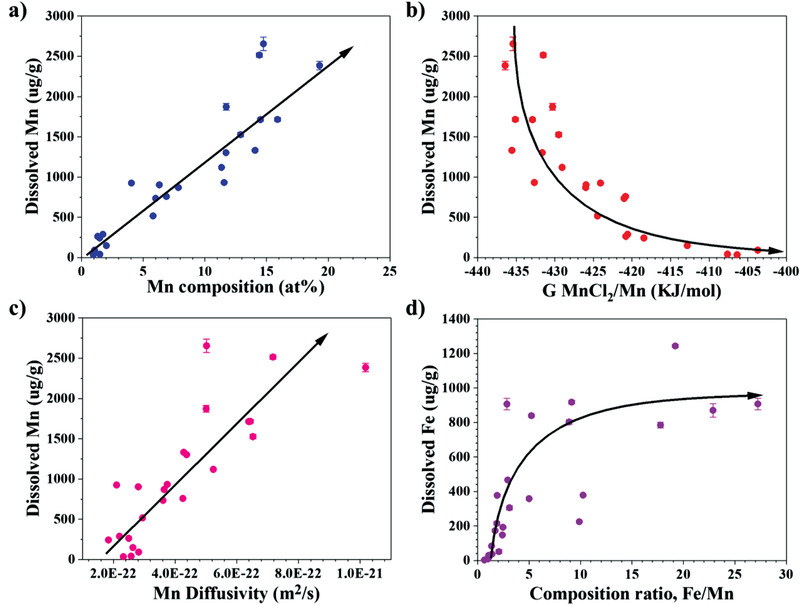
Binary correlations of physical parameters with the dissolutions of Mn and Fe in molten salt (arrow line is added to guide the eye).

Based on the results presented by the RFR model, the two most important factors that affect the corrosion of Mn from the printed alloys, Mn composition and Gibbs free energy of MnCl_2_/Mn, are discussed in greater details. In **Figure** **7**a, the dissolved concentration of Mn is plotted as a function of the Mn composition in the alloy. A clear and expected trend is observed where a higher Mn content in the alloy results in more Mn dissolved in the salt. This means that the dissolution of Mn from the printed alloys gets worse with the increase of Mn content in the alloy. From the perspective of thermodynamics, the MnCl_2_/Mn couple (in different printed alloys) with a lower Gibbs free energy of formation is more likely to have Mn be depleted by molten salt and needs more Mn to dissolve into molten salt such that an equilibrium can be reached between Mn activities in the salt and in the alloy. As a result, an inverse correlation between the dissolved Mn content in molten salt and the Gibbs free energy of MnCl_2_/Mn, calculated by Equation (1), is observed as shown in Figure 7b. The dissolution rate should also be dependent on the elemental diffusion from the bulk to the alloy surface, namely, the diffusivity. The Mn content dissolved in the salt is plotted as a function of its tracer diffusivity in the alloy in Figure 7c and a positive correlation is found. However, the tracer diffusivity was of no importance to Mn dissolution in the ML relative importance ranking order. This means that while the correlation exists (Figure 7c), its importance within the 24 alloys examined in this study is quite weak relative to the other physical parameters, such as the Mn composition and chloride Gibbs free energy of formation.

Fe is the other element that is depleted in the alloy due to corrosion. Unlike Mn, no correlation was observed between the Fe physical parameters and the depletion of Fe from the alloy (see Figure S11, Supporting Information). On the other hand, the Mn related physical parameters, such as Fe/Mn ratio and Mn composition in the alloy, rank at the top in the importance ranking for the depletion of Fe. Thus, the ML indicates that the depletion of Fe will be greatly affected by the behavior of Mn in the printed alloy. A possible mechanism for this interdependency is detailed below based on the concept of “sacrificial protection”. During the corrosion process, Mn atoms at the surface will have the highest driving force to deplete and dissolve into molten salt since the salt redox potential, fixed by the europium redox couple, is higher than that of MnCl_2_,^[^ ^16^, ^30^ ^]^ and the Gibbs free energy of MnCl_2_/Mn is the lowest compared to other three element constituents of the printed alloys (Fe, Cr, and Ni). Following the dissolution, vacancies will be injected in the alloy subsurface (see Figure 4c). From our kinetics results, the diffusivity of Mn in the alloy is two orders of magnitude higher than that of Fe (Figure 5c,d), such that it is much more likely that, if a Mn atom is adjacent to a vacancy, the vacancy will be filled by the Mn atom rather than by an adjacent Fe atom. Consequently, the vacancy flux induced by the atomic dissolution at the surface will result primarily in a Mn flux to the alloy surface by inverse Kirkendall effect.^[^ ^31^ ^]^ This explained why a higher Mn content in the alloy results in more Mn dissolved in the salt as shown in Figure 7c. In other words, and hypothetically, if the diffusivity of Fe is higher than that of Mn, Fe atoms adjacent to the vacancies would fill the vacancies and be further depleted. As such, the Mn could be protected to a certain extent and the alloy having a high Mn content would not necessarily show more Mn depletion.

In the corrosion mechanism of Cr—Fe—Mn—Ni alloys studied, the Mn “sacrifices” itself and protects the Fe from being depleted, even though the Fe is susceptible to corrosion in these conditions, and the Mn composition (i.e., the probability for a Mn atom to be adjacent to a vacancy) ranks the highest in contributing to the elemental dissolution. This is also verified by the correlation between the Fe/Mn ratio in the alloy and the dissolved Fe content as plotted in Figure 7d, implying that the depletion of Fe can be mitigated by increasing the Mn/Fe ratio. The output of the sacrificial protection mechanism is quite analogous to the well‐known sacrificial anode mechanism observed in room temperature aqueous corrosion.^[^ ^32^ ^]^ However, the proposed mechanism is quite unique since it is based on in situ alloying sacrificial mechanism in a homogeneous alloy, rather than a physical separation of the cathode and anode. The above mechanism is also similar to electrochemical de‐alloying,^[^ ^33^ ^]^ but de‐alloying is not a sacrificial protection mechanism because i) it consists of a noble element and a susceptible element (not multiple susceptible elements as in sacrificial protection mechanism) and ii) electrochemical processes are occurring at room temperature, where there would be limited supply of sacrificial atoms at the sample/electrolyte interface by thermal diffusion. To the authors knowledge, this in situ sacrificial alloying during high‐temperature corrosion has not been reported in the past. This unique mechanism is enabled by the active alloying element dissolution in molten salts and the thermally activated diffusion resulting from the high temperature of the environment. While this approach does not lead to a corrosion resistant alloy (i.e., an alloy that would not dissolve any element in aggressive molten salt conditions), it opens new avenue in alloy design for molten salt applications. For instance, an alloying element that is necessary for mechanical properties, but is, on the other hand, electrochemically active, such as Fe, can be protected from dissolution in molten salts by the addition of another electrochemically active and fast diffusing element, such as Mn. Another example would be protecting Cr, which is necessary in high‐temperature alloys to promote the formation of a protective oxide layer at the outer component surface, from dissolving into the salt by alloying with a sacrificial element such as Mn.

## 4 Conclusion

This study developed and utilized a series of innovative HTP experimental and modeling methods to accelerate the discovery of molten salt corrosion‐resistant materials. Starting with the HTP in situ alloying processing, the time savings of using in situ alloying through additive manufacturing for material synthesis can be estimated semi‐quantitatively to be approximately one order of magnitude comparing with the conventional alloy synthesis techniques, such as arc melting of individual samples. When considering the time necessary to homogenize and age arc‐melted versus additively manufactured materials through heat treatment, the total time savings increase to over two orders of magnitude. The details leading to such a reduction in time are highlighted in ref. [13]. Basically, one build plate containing 25 different alloys can be printed in a couple of hours. All the samples on a plate are then polished, homogenized, and aged simultaneously, resulting in considerable time savings relative to regular method (arc melting + quartz ampoule encapsulation of individual samples + polishing). Then, using automated XRD and texture analysis, handheld XRF gun, and automated SEM, while the 25 samples are still attached to the plate, also results in significant time savings in pre‐ and post‐corrosion characterizations. Significant time saving is also resulting from the molten salt droplet tests, which allow the simultaneous corrosion of 25 individual alloys for 96 h using only about 0.37 g of salt for each. Regular corrosion experiments in molten salt would involve i) drilling a small hole in the sample, ii) hanging them to a metal wire through the drilled hole, iii) pour tens of grams of solid salt in a capsule capable of holding only one sample composition to avoid dissimilar materials corrosion effects when different alloys are corroded in the same salt media, iv) locate the capsule into a furnace, note that at this point 25 capsules have to be put and tested one by one in a furnace, and v) take the samples out of each capsule one by one after the corrosion test is over. Steps (i) to (v) are removed using the salt droplet approach developed in the manuscript. Considering the above, we believe that 2 to 3 orders of magnitude in time reduction is a minimum and more time savings could be achieved if all those processes were further optimized. The methodology could also be directly adopted for other alloy systems to accelerate material development.

Using developed integrated HTP approach for the first time, a sacrificial protection mechanism was found in the Cr—Fe—Mn—Ni system, providing new insights on alloy design of structural materials for molten salt applications. For instance, increasing the composition ratio of the unstable element to the less unstable element (Mn/Fe in this study), pending that the diffusivity of the unstable element is relatively higher, can prevent specific elements from dissolving in the salt (Fe in this case). The developed HTP platform, demonstrated in this study, could play a very important role in the rapid development and down‐selection of corrosion‐resistant alloys to support the deployment of molten salt technologies. Although only a limited regime in the Cr—Fe—Mn—Ni compositional space was explored, the HTP platform developed in this study can be used to study the corrosion resistance of alloys in a wide Cr—Fe—Mn—Ni compositional space in future work. This work in combination with future study can be used for machine learning training to predict the corrosion resistance of alloys in the whole regime of Cr—Fe—Mn—Ni compositional space. Finally, it should be noted that the printed alloys investigated in this study exhibited similar microstructures due to the use of the same printing parameters in additive manufacturing process. Using additive manufacturing technologies to modify and tailor the microstructure of printed alloys could be an interesting path forward to explore its effect on the corrosion performance in molten salts.

## 5 Experimental Section

### Additive Manufacturing

In situ alloying via directed‐energy deposition (DED) based additive manufacturing was utilized to manufacture the alloys in a HTP manner. The fabrication was performed in an Optomec LENS MR‐7 system using gas‐atomized elemental powders of Cr, Fe, Mn, and Ni with a size distribution of ≈45–150 µm. Moorehead et al.^[^ ^13^ ^]^ demonstrated the technique of in situ alloying via DED. This process was further modified and refined in this work for the Cr—Fe—Mn—Ni system.

In Optomec LENS MR‐7 system, each powder hopper was filled with one of the elemental powders: Cr, Fe, Mn, or Ni. Flowing Ar gas carried the metal powders from the hoppers into the path of the laser beam. The quantity of the powder to be blown was controlled by the RPM of the augers on the individual powder hoppers. 25 alloy samples, of nominal dimensions 11 mm × 11 mm × 2 mm, were printed on a 316L stainless steel build plate in 5×5 arrays with a spacing of 6.35 mm between samples. Each sample was comprised of five print layers, with two remelting passes after each print layer. The hatch spacing was 0.381 mm for the print layers and 0.19 mm for the remelting layers, with a 90° rotation of the hatch pattern between each subsequent print/remelt layer. Detailed description of the process development can be found in ref. [13].

### Material Preparations and Corrosion Experiments

The LiCl‐KCl eutectic salt used in this study was prepared with 44 wt% of anhydrous LiCl salt powder (≥99% purity, Sigma Aldrich) and 56 wt% of anhydrous KCl salt powder (≥99% purity, Sigma Aldrich). 2 wt% of anhydrous EuCl_3_ (99.99% purity, Sigma Aldrich) was added into LiCl‐KCl eutectic salt to increase the overall corrosion rate by increasing the redox potential of the salt. The LiCl, KCl, and EuCl_3_ salt powders were mixed homogenously according to their corresponding compositions. The trace impurities of the salt mixture were identified by ICP‐MS analysis in which the ten most prominent impurity elements and their concentrations were 16.5 ppm Na,7.5 ppm Mg, 1.1 ppm Al, 3.8 ppm P, 11.2 ppm S, 32.3 ppm Ca, 0.2 ppm Cr, 1.0 ppm Fe, 0.02 ppm Mn, 0.1 ppm Ni. For salt pelletizing, ≈0.375 g salt mixture was weighed, transferred into a custom‐fabricated tungsten carbide die, and compacted through a Pike Pixie Manual Hydraulic Press at a constant load of 2.5 tons for 2 min. The completed salt pill was then punched out by the manual hydraulic press and each salt pill was confirmed to be compact enough as a precaution that ensures minimal mass change of the pill in transit. The entire salt pill preparation process was performed inside an argon atmosphere glovebox (O_2_<2 ppm, H_2_O<0.1 ppm). Before corrosion experiment, the printed alloys were ground with SiC abrasive papers of different grit sizes up to 1200 grit following which they were polished on the polishing pads by 3 µm, 1 µm diamond suspensions, and 0.04 µm colloidal silica suspension. Then the polished alloys were ultrasonically cleaned with deionized water, ethanol, and acetone.

For the corrosion experiment, the pelletized salt pill was placed on the center of the top surface of each printed alloy. Then the salt pill was melted to form a droplet at 500 °C by a heating plate inside the glovebox filled with argon gas. Due to surface tension, the molten salt droplet stayed on the surface of the printed alloy without spillage. The corrosion experiment was performed for 96 h after which the heating plate was shut down and the entire solidified salt droplet was removed from each of the printed alloys for further analysis. A low‐speed saw was used to cut the post‐corrosion samples of which material characterizations were performed on the cross sections. The cross sections were then polished using the same procedure as done for the printed alloys before the corrosion experiment.

### Material Characterizations

SEM coupled with EDS and EBSD was performed at the Wisconsin Center for Nanoscale Technologies to characterize the samples before and after corrosion experiment. EBSD was acquired at a magnification of 100 times and ≈14 mm working distance using a step size of 5 µm. Before analysis, the acquired EBSD data was cleaned by eliminating grains with diameters less than 5 µm and misorientations less than 0.5°. XRD used in this study was conducted by Bruker D8 Discover diffractometer with a Cu‐K*α* micro X‐ray source. XRD pole figure was normalized by an internal Bruker software and the normalized data were exported and plotted using MTEX software after background and defocusing correction. There were still some artifacts in the XRD pole figure that could be a result from the florescence and/or a sector of the detector that has lower intensity. GDOES was used to provide information regarding the concentration profile of each element along the depth of the analyzed sample through which the depletion depth could be identified without the need to cut and polish the post‐corrosion samples as done in traditional material characterization approaches. The GDOES was carried out by Horiba GD‐profiler 2 using a pressure of 550 Pa and a power of 40 W for the plasma generation. The calibration of GDOES was performed using sputtering rate correction with certified materials with known compositions which covered the concentration ranges of ≈0–100 wt% for Ni and Fe while ≈0–30 wt% for Cr and Mn, respectively. The standard deviation of the measured data was derived based on the uncertainty on the concentrations of the standards, sputtering rate of the standards, the detection limit, and the measured intensity of the standards using classical error propagation formulas. The measured GDOES data was analyzed by a developed algorithm as follows to obtain the depletion depth:

1) Determining an average value $\bar{C}$ based on the bulk composition data of the depleted element as displayed at the right side of the GDOES profile window where the composition curves were stable. Calculating the corresponding uncertainty *σ* based on the 95% confidence interval from the calibration curves, as displayed by the shaded area in GDOES profile.
2) Searching the composition data *C_d_* from the sample surface (depth is 0) verifying $C_{d}=\bar{C}\;-\sigma$ on the composition curve, the upper uncertainty curve, and the lower uncertainty curve, respectively. Thus, the depletion depth was defined as the point at which the measured concentration deviates from the 95% confidence interface of the bulk concentrations.
3) The average value of the three corresponded depths of *C*_d_ was defined as the corrosion attack depth (depletion depth) and the standard deviation of these three corresponded depths was added as errors bars in Figure 4a.

Except alloy #5, alloy #7, alloy #17, and alloy #20 for which the depletion depth was measured by EDS line scan, all other alloys’ depletion depths were obtained from GDOES analysis. For alloy #5, alloy #7, alloy #17, and alloy #20, three EDS line scans were conducted at different locations of each sample, the final depletion depth was defined based on the average value of the depletion depths obtained from the three EDS line scans and the standard deviation was used as the error bar as shown in Figure 4a. Previous study directly comparing the element variation depths of two Ni‐201 alloy at the same experiment condition measured by GDOES and EDS had shown similar results from both techniques,^[^ ^17^ ^]^ therefore, validating both techniques for determining the corrosion resistance of additive manufactured alloys in this study. However, it still needs to be noted that GDOES sputtered area included tens of thousands of grains such that the depletion depth measured by GDOES was a convolution of uniform and localized corrosion, if any. The deepest corrosion attack feature might not be characterized by GDOES. Dissolved cations in post‐corrosion salt were quantified by digesting the whole solidified salt droplet after corrosion experiment into deionized water and then analyzing it using ICP‐MS at the Wisconsin State Laboratory of Hygiene. One benefit of using salt pills in the corrosion experiments was that the entire pill can be dissolved for ICP‐MS analysis, ensuring the measured analyte concentration in the sample matrix represented the averaged composition of the entire salt volume participating in the corrosion process.

### DFT and CALPHAD Modeling

Surface energies and work functions of various FCC alloy compositions were calculated by DFT, implemented by the Vienna Atomistic Simulation Package (VASP).^[^ ^34^ ^]^ The considered compositions ranged from unary to quaternary with a composition step of 25 at% for binary and ternary and 12.5% for quaternary. Random alloy structures were modeled by 32‐atom special quasi‐random structures (SQS)^[^ ^35^ ^]^ using the ATAT package^[^ ^36^ ^]^ while surface structures were generated from the SQSs using the Pymatgen package.^[^ ^37^ ^]^ Generalized gradient approximation (PBE) projector augmented wave pseudopotential^[^ ^38^ ^]^ was used to describe the exchange‐correlation contribution and collinear spin polarization was accounted. Integration in the reciprocal space were obtained over a gamma‐centered Monkhorst–Pack grid with N_kpoint_ ≈ 3000/N_atom_ within the first Brillouin zone.^[^ ^39^ ^]^ Cut‐off energy was set to the 1.3 times of the highest constitutional ENMAX. The electronic and ionic convergence criteria were 10^−6^/10^−5^ eV respectively for bulk calculations while 10^−5^/10^−4^ eV respectively for surface calculations. The bulk structures’ atomic positions, volumes, and shapes were fully relaxed while only atomic positions were considered for the relaxations of surface structures. These relaxations were achieved by Hermite–Gauss smearing method of Methfessel and Paxton of order 1, with a smearing parameter of 0.01 eV.^[^ ^40^ ^]^ At the end, Final static calculations using the tetrahedron smearing method with Blöchl corrections^[^ ^41^ ^]^ were conducted for all calculations to improve the accuracy. The calculation process was detailed in ref. [21].

The calculated surface energies and work functions of the considered FCC compositions were then used to assess CALPHAD sub‐regular solution model which allowed the interpolation of data at arbitrary compositions within the quaternaries. Thus, the surface energy and work function of the printed alloy compositions could be derived based on the fitted CALPHAD model. The CALPHAD sub‐regular solution model can be expressed by

(2) $$P\;=\sum_{i}x_{i}\;+\sum_{i}\sum_{j\neq i}a_{ij}x_{i}x_{j}+\sum_{i}\sum_{j\neq i}\sum_{k\neq i,j}a_{ijk}x_{i}x_{j}x_{k}$$

where, *P* is either surface energy or work function, *x*_*i*,*j*, *k*_ is the composition of Cr, Fe, Mn, or Ni, *a_ij_* and *a_ijk_* were model parameters that were derived using the calculated surface energies and work functions. To account for the CALPHAD model's uncertainty, quantification approach based on Bayesian statistics was also adopted and the details of which can be found in literature.^[^ ^42^ ^]^

### Machine Learning

A vector of 15 parameters including compositions, Fe/Mn composition ratio, Gibbs free energy, self‐diffusivity, activity of Mn, Fe, and Ni, mean surface energy and work function parameterizing physical properties was associated to each alloy tested (“physical descriptors/features” of the alloy). These physical descriptors were curated by considering physical parameters of an alloy which were theoretically expected to directly or indirectly influence corrosion behavior of the alloy regardless of whether their relationships to target variables were explicitly known. Upon completion of the corrosion experiment, the corrosion performance of the alloy was parameterized by two “target variables”: the concentrations of corroded Fe and Mn into the post‐corrosion salt. A sample set containing 28 alloys (4 alloys are from repeat test), each with 15 descriptors and 2 target variables was used to train a random Forest regressor (RFR) model using the Scikitlearn package.^[^ ^26^ ^]^ The RFR model was hyperparameter‐tuned to optimize the number of estimators (1000), and maximum tree depth (none). The target variables were predicted with this RFR model using fivefold cross‐validation (CV) to safeguard against overfitting.^[^ ^29^ ^]^ The ranking of features by importance score was obtained from the RFR model, indicating the relative significance of variables that influence the corrosion outcome measured by target variable value, with the most influential variable ranked at the top. This ranking order was found to be reproducible with a variation in random seed. The RFR model was retrained with a series of subsets of the original feature set and the relative feature rankings were found to be reproducible as well. This indicated robustness of the model and reliability of the ranking order.

### Statistical Analysis

Raw data were directly used in statistical analysis with no data excluded. Data were present as mean ± s.d. The sample sizes (*n*) and *p* values were specified in the figure captions.

## Conflict of Interest

The authors declare no conflict of interest.

## Author Contributions

Y.W. constructed the experimental facility with the assistance from B.G.; Y.W. performed corrosion experiments, data analysis, and sample preparations; Y.W., P.N., R.D., and, N. H. conducted the material characterizations; P.N. and M.M. performed additive manufacturing; B.G. and J.H. contributed the machine learning; Y.W. and T.D. performed the thermodynamic/kinetic modeling; Y.W. and A.C. drafted the manuscript; A.C., K.S., D.T., and S.C. conceived of the original project and oversaw its execution, providing regular guidance.

## Supporting information

Supporting Information is available from the Wiley Online Library or from the author.

## Data Availability

The data that support the findings of this study are available in the supplementary material of this article.

## References

[1] C. Forsberg , L.‐w. Hu , P. Peterson , K. Sridharan , Fluoride‐Salt‐Cooled High‐Temperature Reactor (FHR) Commercial Basis and Commercialization Strategy, Massachusetts Institute of Technology, Cambridge, MA 2015.

[2] J. J. Laidler , J. E. Battles , W. E. Miller , J. P. Ackerman , E. L. Carls , Prog. Nucl. Energy 1997, 31, 131.

[3] J. C. Gomez‐Vidal , . Tirawat , Sol. Energy Mater. Sol. Cells 2016, 157, 234.

[4] T. Ouchi , H. Kim , B. L. Spatocco , R. Sadoway , Nat. Commun. 2016, 7, 10999. 27001915 10.1038/ncomms10999 PMC4804165

[5] M. Elbakhshwan , W. Doniger , C. Falconer , M. Moorehead , C. Parkin , C. Zhang , K. Sridharan , A. Couet , Sci. Rep. 2019, 9, 18993. 31831873 10.1038/s41598-019-55653-2 PMC6908586

[6] W. Ren , in ASME 2018 Pressure Vessels and Piping Conference, American Society of Mechanical Engineers, New York 2018, pp. 1–9.

[7] R. N. Wright , T.‐L. Sham , Status of Metallic Structural Materials for Molten Salt Reactors, Argonne National Lab, Argonne, IL 2018.

[8] J. Sha , H. Hirai , T. Tabaru , A. Kitahara , H. Ueno , S. Hanada , Mater. Sci. Eng., A 2004, 364, 151.

[9] O. Guillon , J. Gonzalez‐Julian , B. Dargatz , T. Kessel , G. Schierning , J. Räthel , M. Herrmann , Adv. Eng. Mater. 2014, 16, 830.

[10] K. Sridharan , T. R. Allen , In Molten Salts Chemistry, Elsevier, New York 2013.pp. 241–267

[11] A. G. Fernández , M. I. Lasanta , F. J. Pérez , Oxid. Met. 2012, 78, 329.

[12] C. Atwood , M. Griffith , L. Harwell , E. Schlienger , M. Ensz , J. Smugeresky , T. Romero , D. Greene , D. Reckaway , Laser Engineered Net Shaping (LENS): A Tool for Direct Fabrication of Metal Parts, Laser Institute of America, Orlando, IL 1998.

[13] M. Moorehead , K. Bertsch , M. Niezgoda , C. Parkin , M. Elbakhshwan , K. Sridharan , C. Zhang , D. Thoma , A. Couet , Mater. Des. 2020, 187, 108358.

[14] M. Moorehead , P. Nelaturu , M. Elbakhshwan , C. Parkin , C. Zhang , K. Sridharan , D. J. Thoma , A. Couet , J. Nucl. Mater. 2021, 547, 152782.

[15] P. Agrawal , S. Gupta , S. Thapliyal , S. Shukla , R. S. Haridas , R. S. Mishra , Addit. Manuf. 2020, 37, 101623.

[16] S. Guo , W. Zhuo , Y. Wang , J. Zhang , Corros. Sci. 2020, 163, 108279.

[17] C. Falconer , W. H. Doniger , L. Bailly‐Salins , E. Buxton , M. Elbakhshwan , K. Sridharan , A. Couet , Corros. Sci. 2020, 177, 108955.

[18] J. Park , S. Choi , S. Sohn , I. S. Hwang , J. Electrochem. Soc. 2017, 164, D744.

[19] D. Inman , J. C. L. Legey , R. Spencer , J. Electroanal. Chem. Interfacial Electrochem. 1975, 61, 289.

[20] D. Horvath , D. Rappleye , P. Bagri , M. F. Simpson , J. Nucl. Mater. 2017, 493, 189.

[21] T. Duong , Y. Wang , X. Yan , A. Couet , S. Chaudhuri , arXiv:2104.10590 2021.

[22] H. Ma , X.‐Q. Chen , R. Li , S. Wang , J. Dong , W. Ke , Acta Mater. 2017, 130, 137.

[23] C. Zhang , F. Zhang , K. Jin , H. Bei , S. Chen , W. Cao , J. Zhu , D. Lv , J. Phase Equilib. Diffus. 2017, 38, 434.

[24] C. Li , J. Yin , K. Odbadrakh , B. C. Sales , S. J. Zinkle , G. M. Stocks , B. D. Wirth , J. Appl. Phys. 2019, 125, 155103.

[25] Y. Wang , K. Sridharan , A. Couet , J. Nucl. Mater. 2021, 543, 152624.

[26] L. Breiman , Mach. Learn. 1996, 24, 123.

[27] H. D. White , J. Am. Soc. Inf. Sci. Technol. 2003, 54, 1250.

[28] M. Jin , P. Cao , P. Short , J. Nucl. Mater. 2019, 523, 189.

[29] B. Meredig , E. Antono , C. Church , M. Hutchinson , J. Ling , S. Paradiso , B. Blaiszik , I. Foster , B. Gibbons , J. Hattrick‐Simpers , A. Mehta , L. Ward , Mol. Syst. Des. Eng. 2018, 3, 819.

[30] D. Horvath , D. Rappleye , P. Bagri , M. F. Simpson , J. Nucl. Mater. 2017, 493, 189.

[31] A. D. Smigelskas , Trans. AIME 1947, 171, 130.

[32] I.‐C. Park , S.‐J. Kim , Appl. Surf. Sci. 2020, 509, 145346.

[33] E. A. Brandes , G. B. Brook , Smithells Metals Reference Book, Elsevier, New York 2013.

[34] G. Kresse , J. Furthmüller , Phys. Rev. B 1996, 54, 11169. 10.1103/physrevb.54.11169 9984901

[35] A. Zunger , S.‐H. Wei , L. G. Ferreira , J. E. Bernard , Phys. Rev. Lett. 1990, 65, 353. 10042897 10.1103/PhysRevLett.65.353

[36] A. van de Walle , R. Sun , Q.‐J. Hong , S. Kadkhodaei , Calphad 2017, 58, 70.

[37] R. Tran , Z. Xu , B. Radhakrishnan , D. Winston , W. Sun , K. A. Persson , S. P. Ong , Sci. Data 2016, 3, 160080. 27622853 10.1038/sdata.2016.80 PMC5020873

[38] J. P. Perdew , K. Burke , M. Ernzerhof , Phys. Rev. Lett. 1996, 77, 3865. 10062328 10.1103/PhysRevLett.77.3865

[39] H. J. Monkhorst , J. D. Pack , Phys. Rev. B 1976, 13, 5188.

[40] M. P. A. T. Methfessel , A. T. Paxton , Phys. Rev. B 1989, 40, 3616. 10.1103/physrevb.40.3616 9992329

[41] P. E. Blöchl , O. Jepsen , O. K. Andersen , Phys. Rev. B 1994, 49, 16223. 10.1103/physrevb.49.16223 10010769

[42] T. C. Duong , R. E. Hackenberg , A. Landa , P. Honarmandi , A. Talapatra , H. M. Volz , A. Llobet , A. I. Smith , G. King , S. Bajaj , A. Ruban , L. Vitos , P. E. A. Turchi , R. Arróyave , Calphad 2016, 55, 219.

