# Supplementary material for: Integrated High‐Throughput and Machine Learning Methods to Accelerate Discovery of Molten Salt Corrosion‐Resistant Alloys
Source: Adv Sci (Weinh). 2022 May 7;9(20):2200370. doi: 10.1002/advs.202200370 (PMC9284150; doi:10.1002/advs.202200370)
Supplement: Supplementary file 1 — Supporting Information is available from the Wiley Online Library or from the author. [file ADVS-9-2200370-s001.pdf]

ADVANCED SCIENCE
Open Access

Supporting Information

for *Adv. Sci.*, DOI 10.1002/advs.202200370

Integrated High-Throughput and Machine Learning Methods to Accelerate Discovery of Molten Salt Corrosion-Resistant Alloys

Yafei Wang, Bonita Goh, Phalgun Nelaturu, Thien Duong, Najlaa Hassan, Raphaelle David, Michael Moorehead, Santanu Chaudhuri, Adam Creuziger, Jason Hattrick-Simpers, Dan J. Thoma, Kumar Sridharan and Adrien Couet*

Supporting Information

Table S1. Compositions of the 25 printed alloys identified by EDS and XRF.

sample number	EDS compositions (at%)				XRF compositions (at%)			
#	Cr	Fe	Mn	Ni	Cr	Fe	Mn	Ni
1	0.5	6.0	0.3	93.2	0.7	3.2	0.5	95.6
2	0.6	10.4	1.1	87.9	0.5	9.8	0.6	89.1
3	0.8	13.8	12.9	72.5	1.1	10.5	9.9	78.5
4	0.8	13.1	6.9	79.3	0.9	11.0	5.3	82.8
5	1.3	17.1	6.0	75.6	0.8	15.1	5.6	78.5
6	1.2	18.4	2.0	78.4	1.1	16.8	1.6	80.5
7	2.4	13.2	1.5	82.9	4.2	12.1	0.8	83.0
8	2.6	17.0	1.0	79.5	4.4	16.1	0.6	78.8
9	1.3	12.6	19.3	66.7	1.6	9.8	15.4	73.2
10	0.9	19.6	14.5	65.0	1.0	16.5	10.6	72.0
11	0.6	14.8	14.4	70.1	0.9	11.7	12.0	75.3
12	1.0	22.0	11.7	65.3	0.5	20.2	9.5	69.8
13	0.7	19.2	11.4	68.7	0.9	17.0	8.4	73.7
14	0.8	29.0	5.8	64.4	0.4	26.0	4.7	68.8
15	1.1	24.3	7.8	66.8	0.6	21.9	5.1	72.4
16	0.6	33.3	1.7	64.3	0.8	31.6	0.9	66.7
17	1.1	30.0	1.3	67.6	0.4	28.0	0.7	70.9
18	1.3	22.3	15.9	60.5	0.7	21.1	10.8	67.5
19	0.9	28.1	11.7	59.2	0.6	26.6	8.7	64.1
20	0.9	32.9	6.3	59.8	0.9	31.6	5.4	62.2
21	0.9	30.5	14.7	53.9	1.7	26.7	10.8	60.8
22	1.3	33.8	11.6	53.4	0.5	32.7	8.2	58.5
23	0.8	41.5	4.0	53.7	0.8	36.1	4.4	58.7
24	0.9	40.1	1.5	57.5	0.6	38.6	1.5	59.4
25	1.1	34.7	14.1	50.1	0.8	31.2	12.3	55.7

Table S2. Lattice constant of the 25 different printed alloys.

sample number	Lattice constant	error
#	Å	Å
1	3.528	1.32E-04
2	3.537	1.28E-04
3	3.563	2.33E-04
4	3.550	1.62E-04
5	3.555	1.73E-04
6	3.546	1.48E-04
7	3.542	1.10E-04
8	3.544	1.41E-04
9	3.576	2.42E-04
10	3.572	2.29E-04
11	3.574	2.35E-04
12	3.572	2.60E-04
13	3.565	2.45E-04
14	3.570	2.70E-04
15	3.564	2.26E-04
16	3.564	2.57E-04
17	3.559	2.41E-04
18	3.579	2.61E-04
19	3.567	2.40E-04
20	3.580	3.06E-04
21	3.587	2.72E-04
22	3.581	3.00E-04
23	3.579	3.44E-04
24	3.575	2.91E-04
25	3.595	2.94E-04

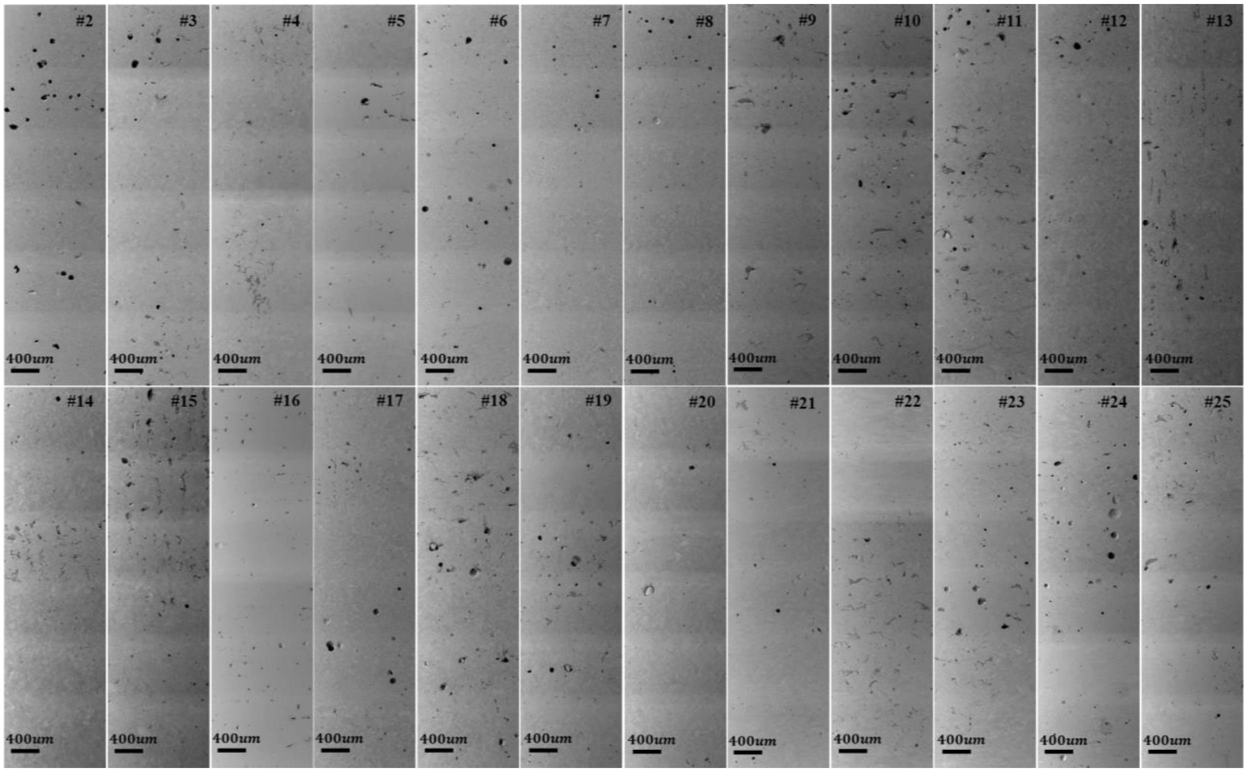

Figure S1. SEM images of the printed alloys before corrosion experiment at lower magnification.

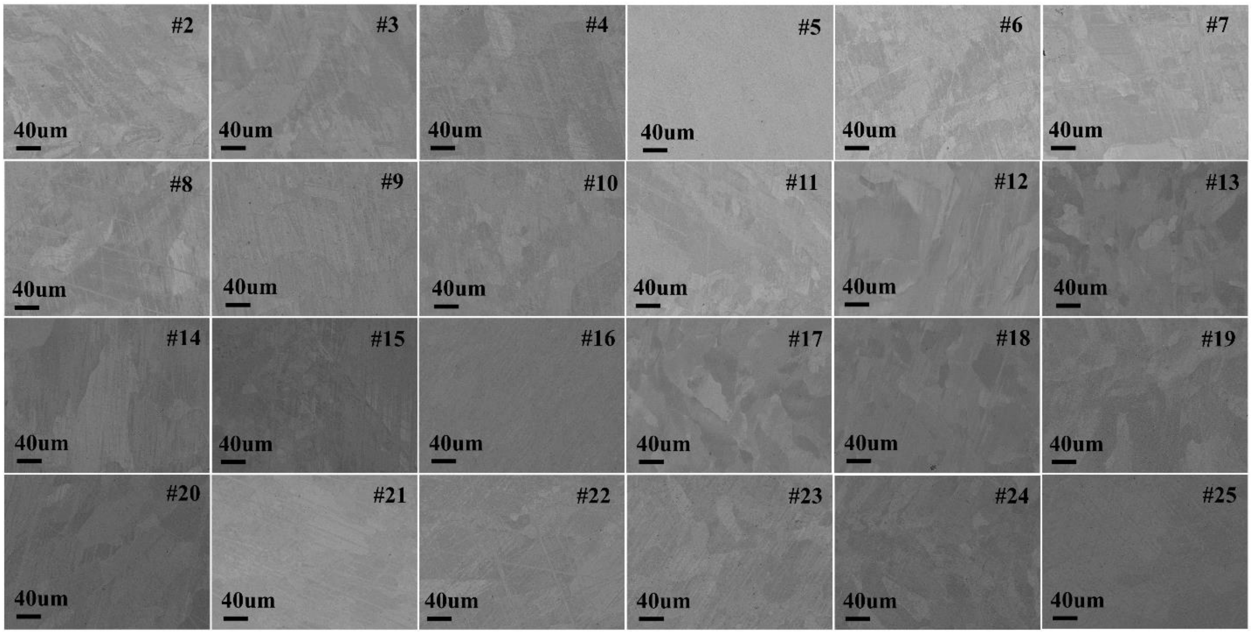

Figure S2. SEM images of the printed alloys before corrosion experiment at higher magnification.

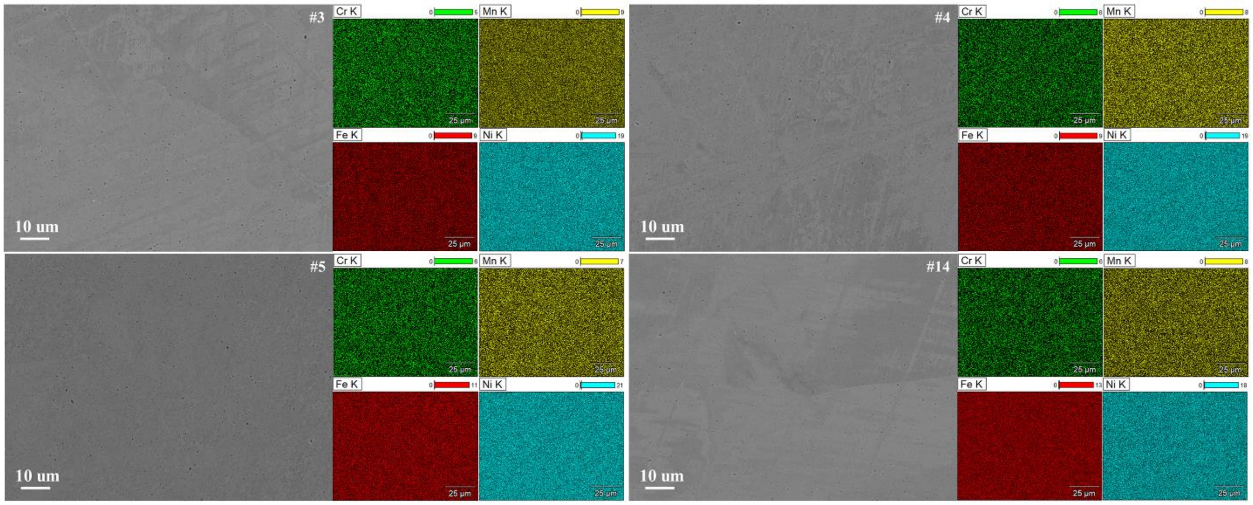

Figure S3. EDS elemental mappings of a few representative printed alloys.

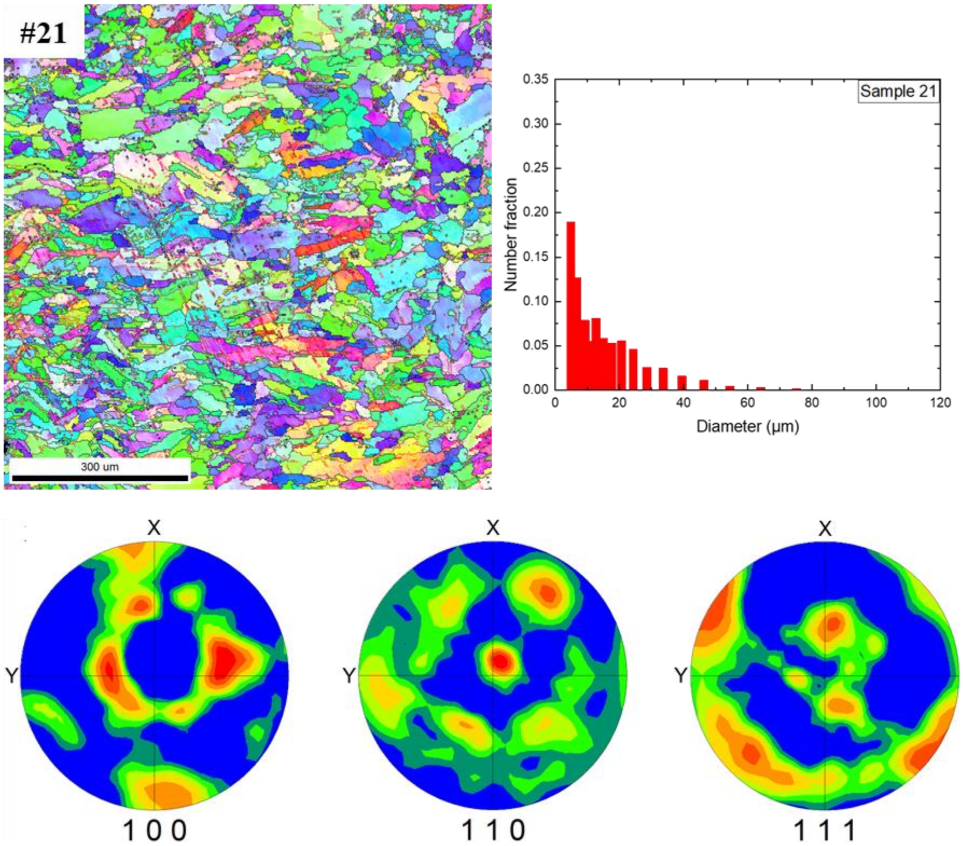

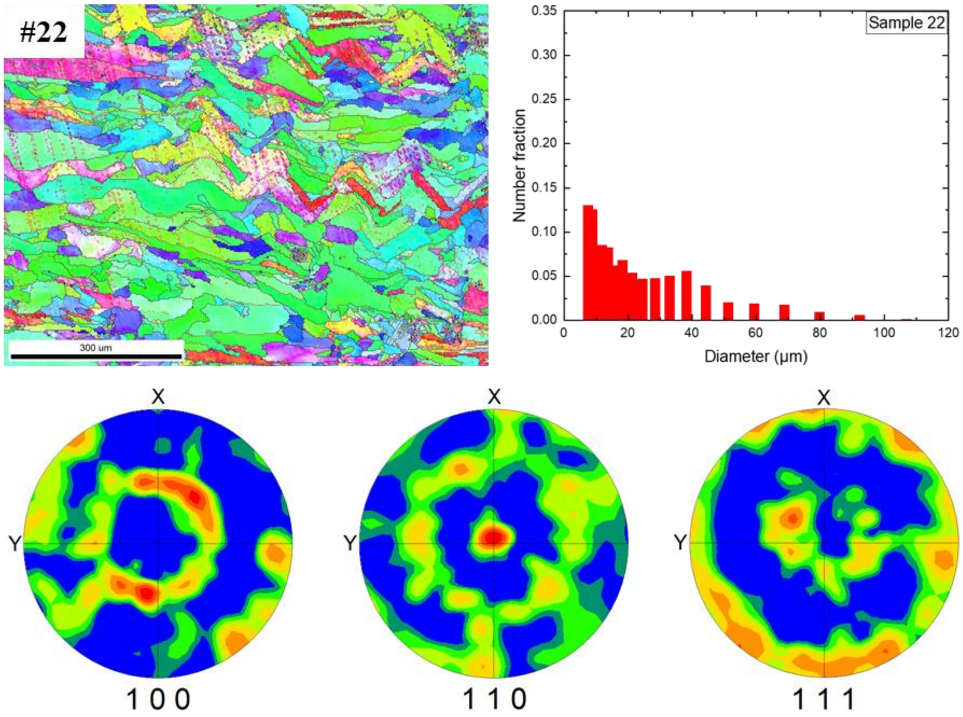
#22
300 um
Sample 22
Number fraction
Diameter (μm)
0.35
0.30
0.25
0.20
0.15
0.10
0.05
0.00
0
20
40
60
80
100
120
X
Y
1 0 0
1 1 0
1 1 1

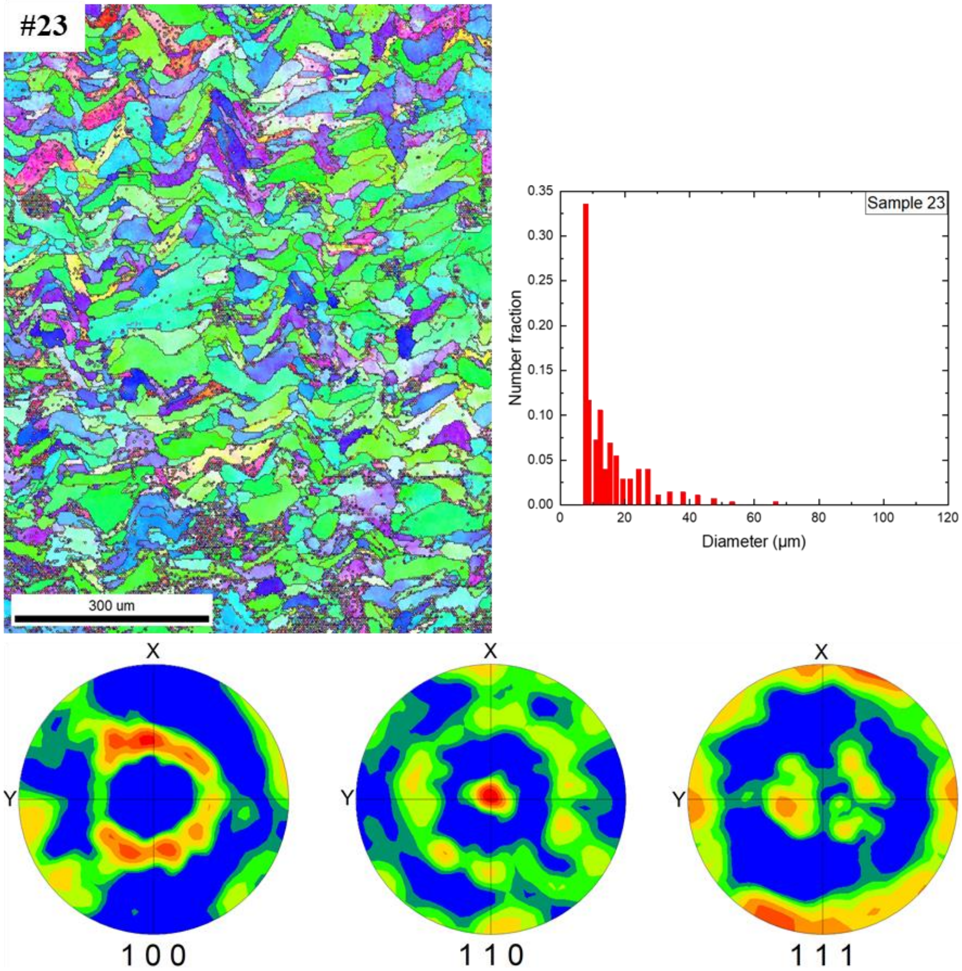
#23
300 um
Sample 23
Number fraction
Diameter (μm)
0.35
0.30
0.25
0.20
0.15
0.10
0.05
0.00
0
20
40
60
80
100
120
X
Y
1 0 0
1 1 0
1 1 1

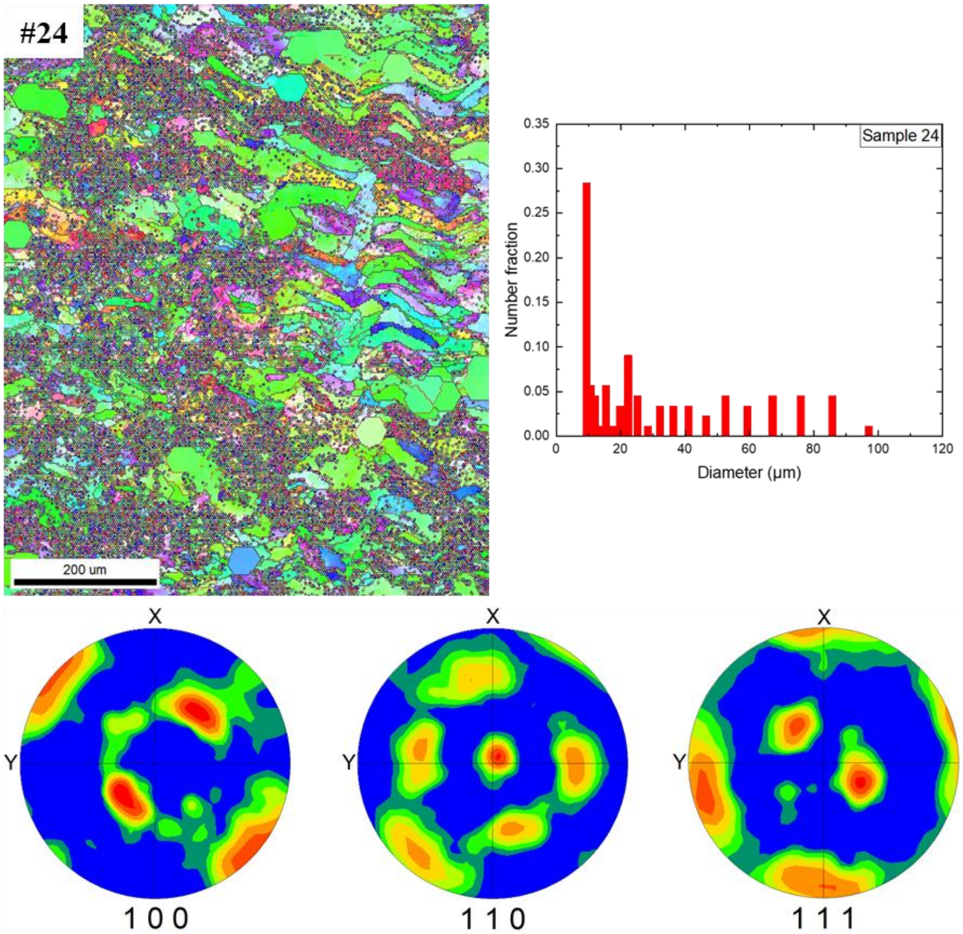
#24
200 um
Sample 24
Number fraction
Diameter (μm)
0.00
0.05
0.10
0.15
0.20
0.25
0.30
0.35
0
20
40
60
80
100
120
X
Y
1 0 0
1 1 0
1 1 1

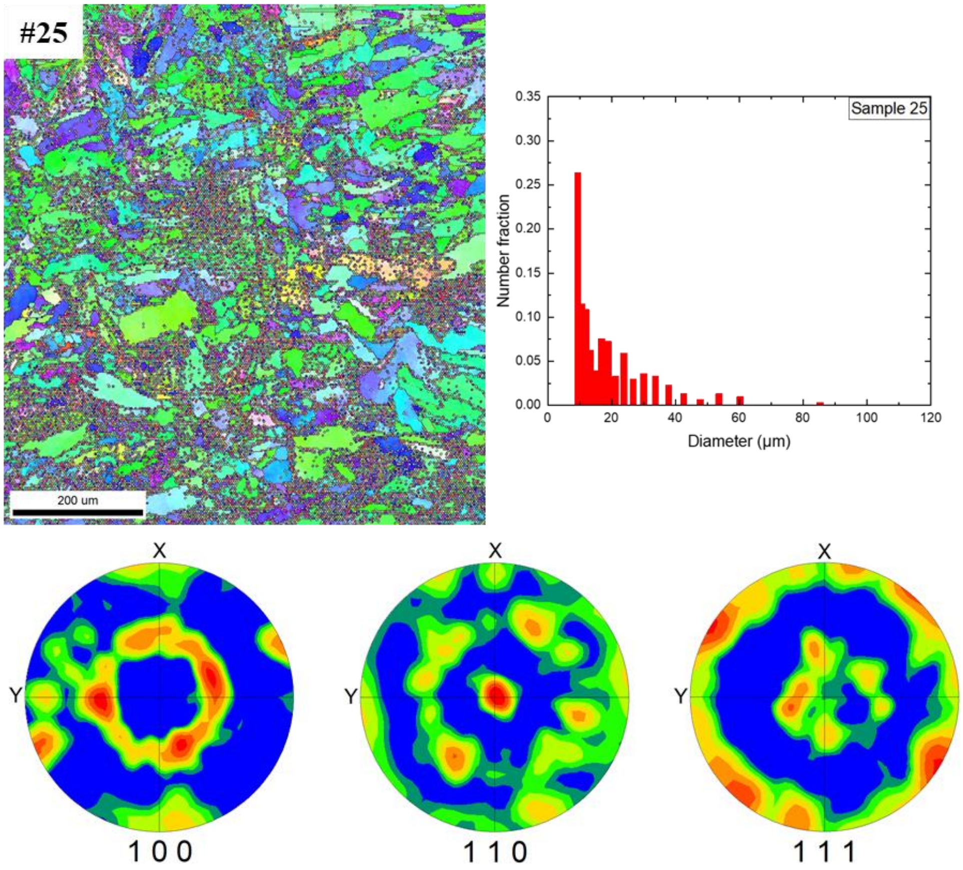

Figure S4. EBSD IPF maps, grain size distributions, and pole figures of alloys #21, #22, #23, #24, and #25.

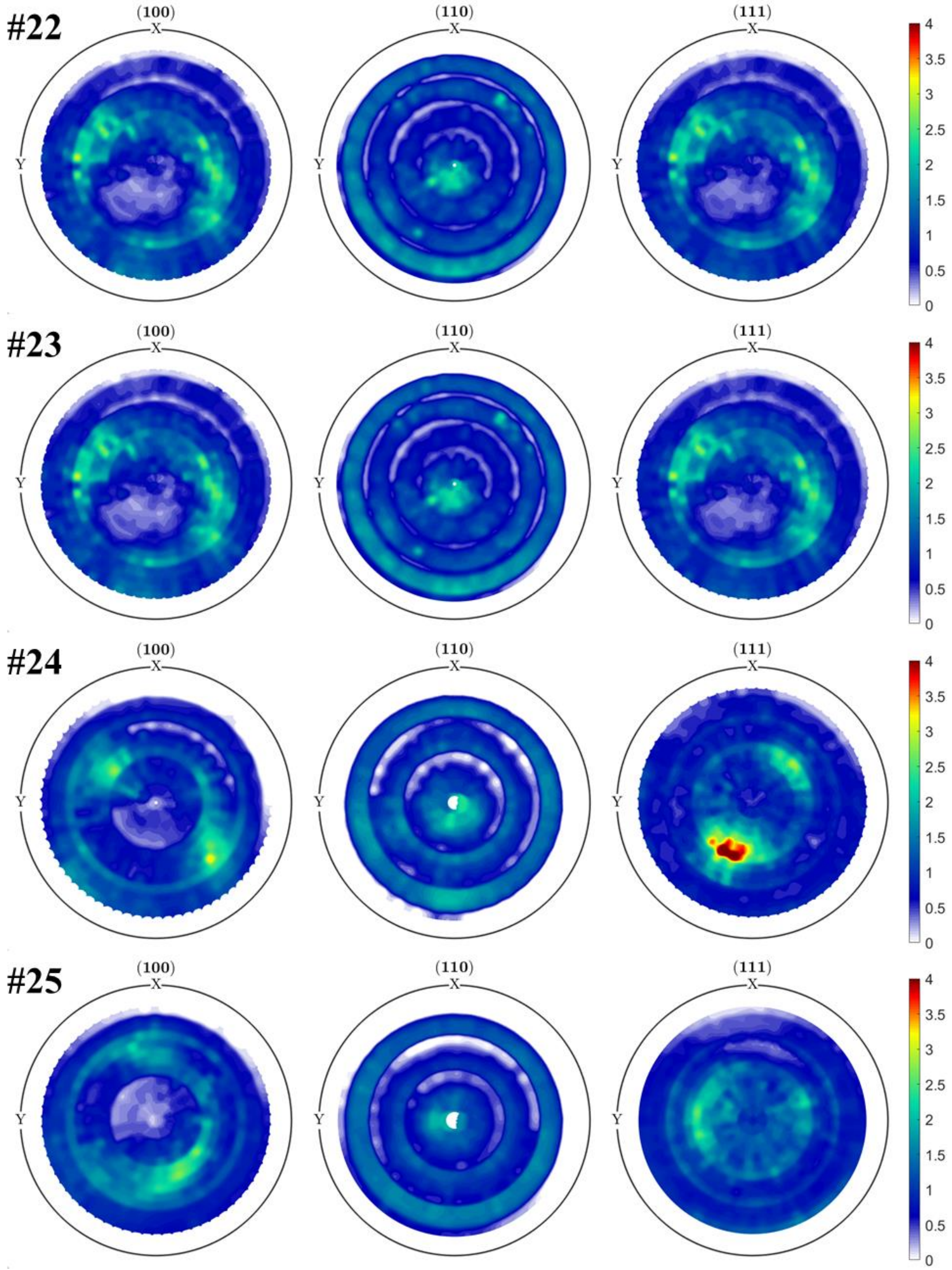

Figure S5. XRD pole figures of alloys #22, #23, #24, and #25.

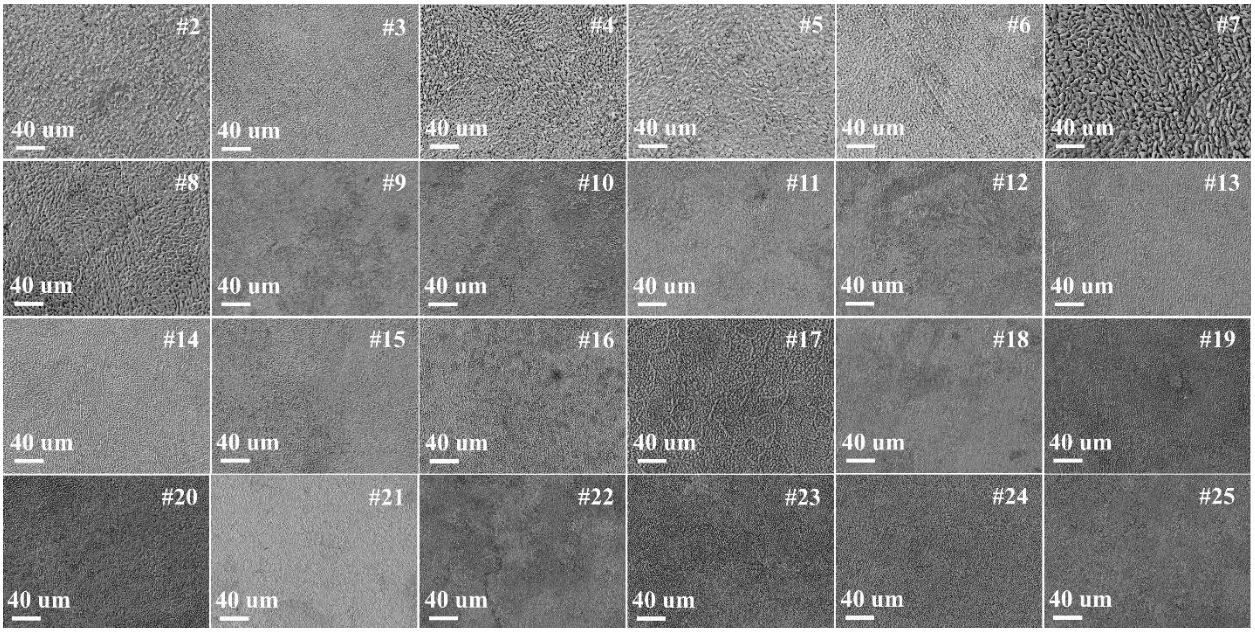

Figure S6. SEM images of the printed alloys after corrosion experiment at lower magnification.

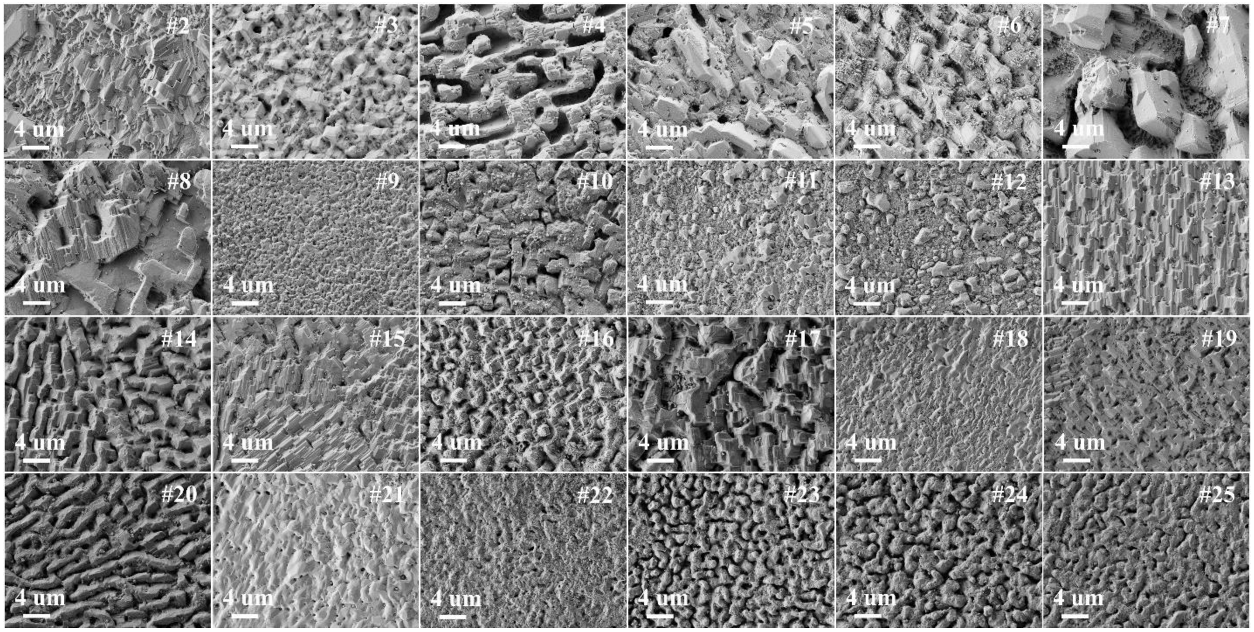

Figure S7. SEM images of the printed alloys after corrosion experiment at higher magnification.

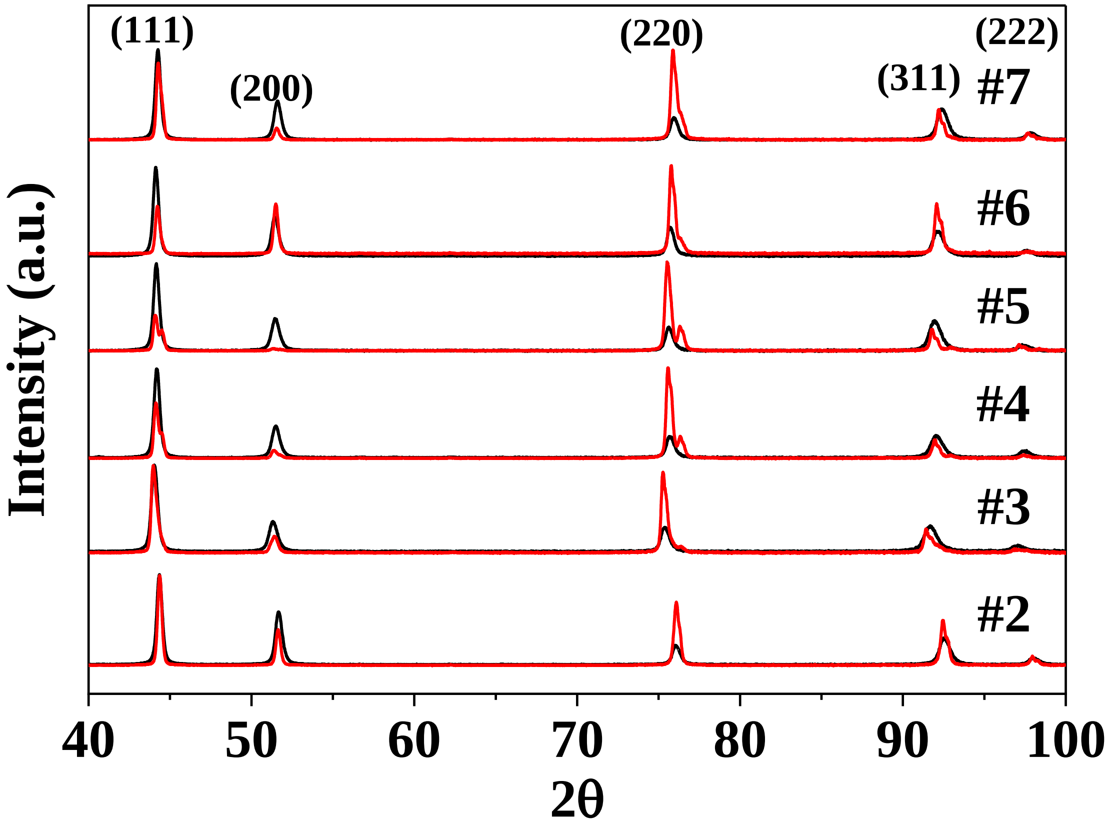
(111)
(200)
(220)
(311)
(222)
#7
#6
#5
#4
#3
#2
Intensity (a.u.)
40
50
60
70
80
90
100
2θ

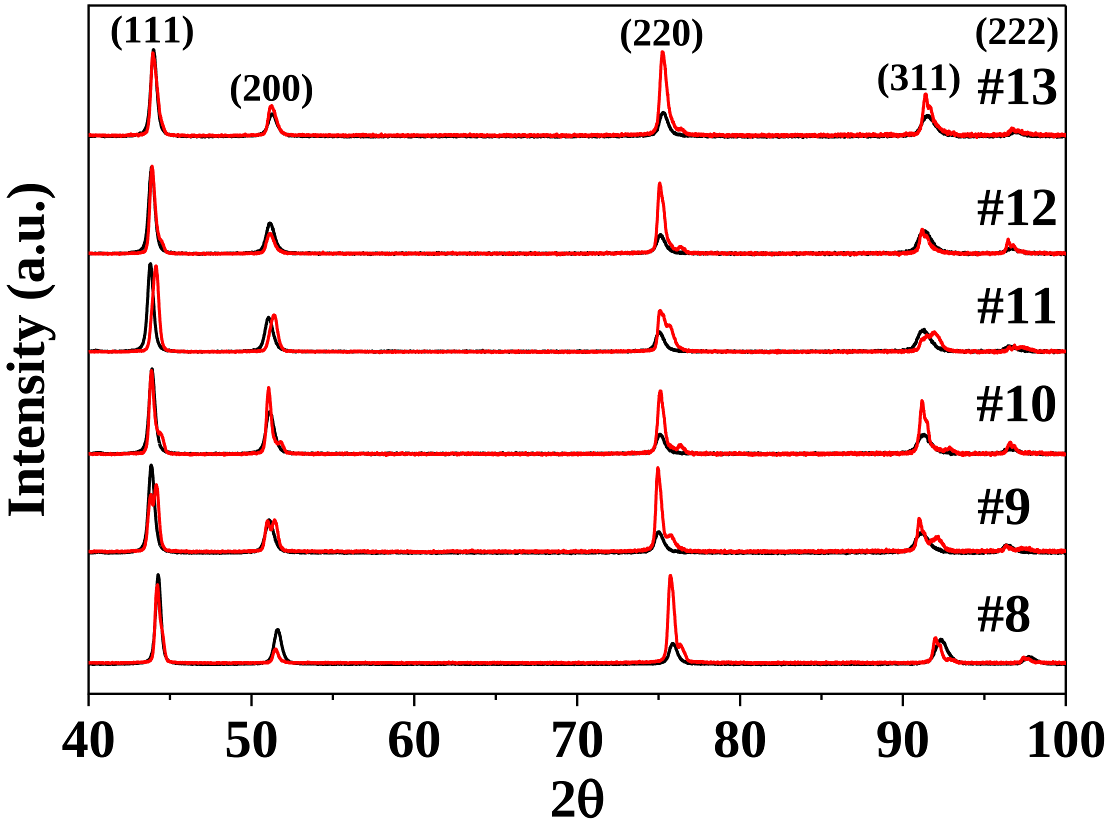
(111)
(200)
(220)
(311)
(222)
#13
#12
#11
#10
#9
#8
Intensity (a.u.)
40
50
60
70
80
90
100
2θ

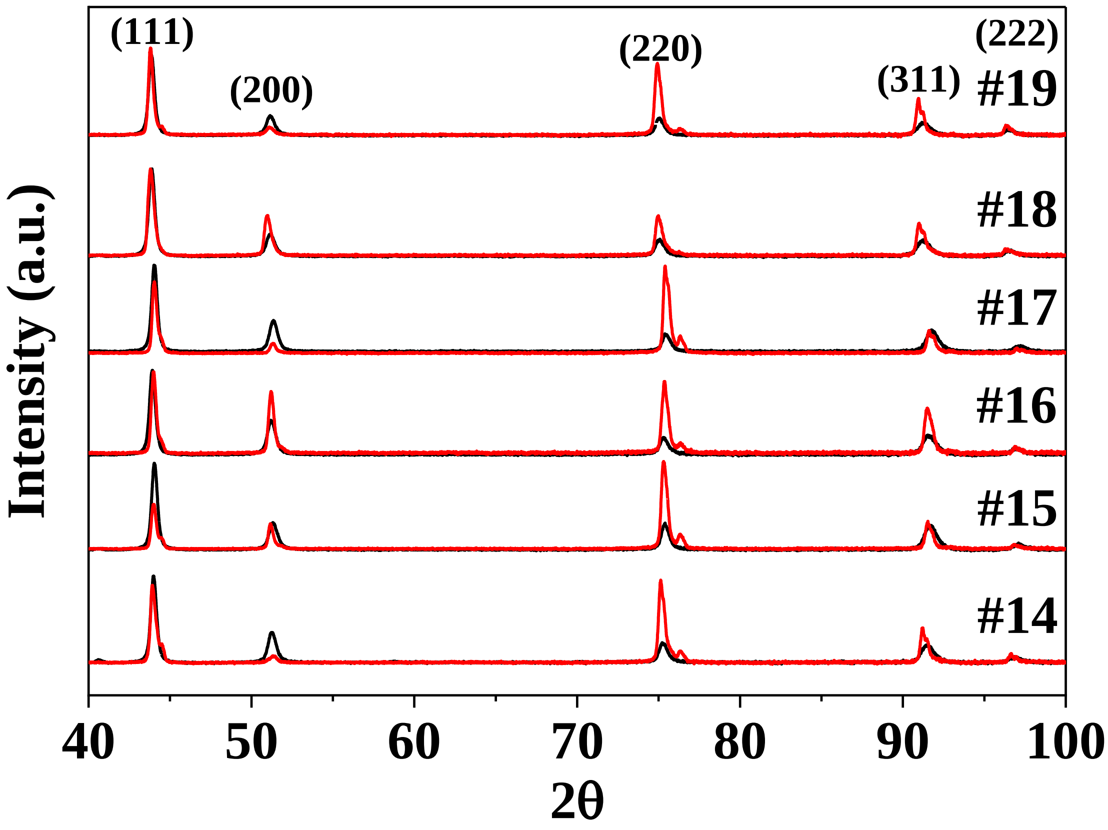
(111)
(200)
(220)
(311)
(222)
#19
#18
#17
#16
#15
#14
Intensity (a.u.)
40
50
60
70
80
90
100
2θ

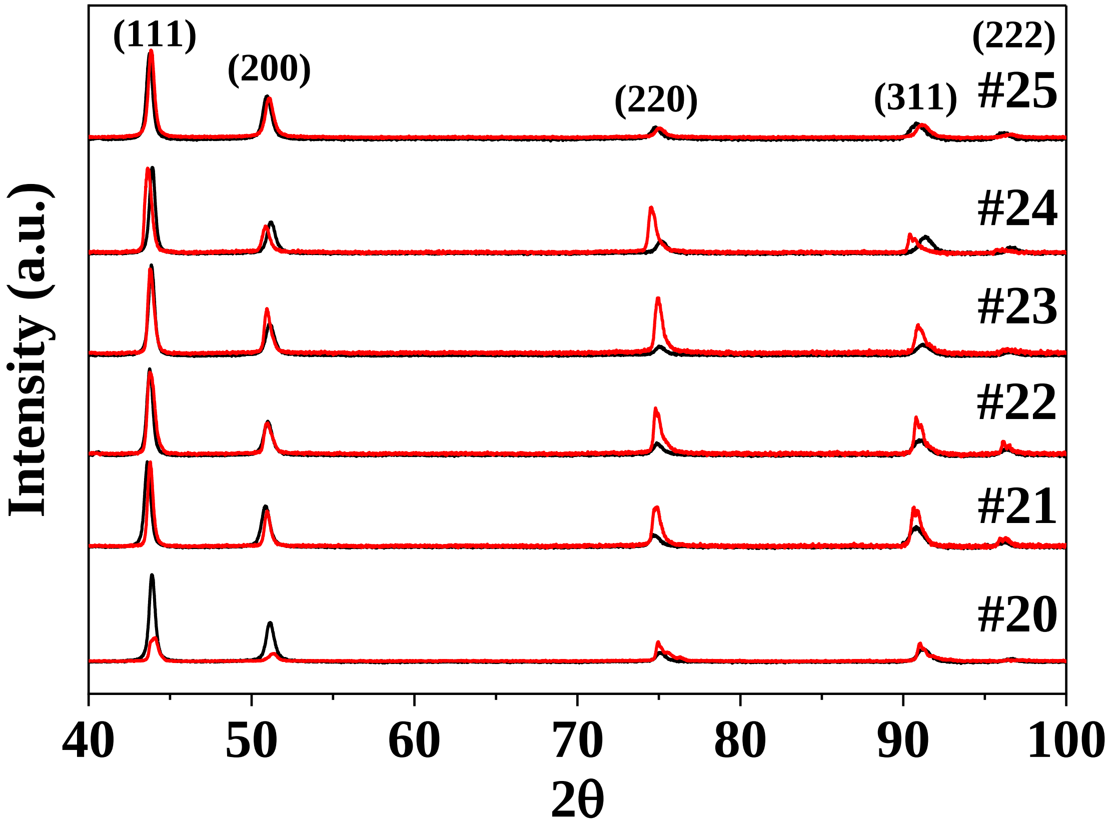

Figure S8. XRD patterns of the printed alloys before (black curves) and after (red curves) corrosion test.

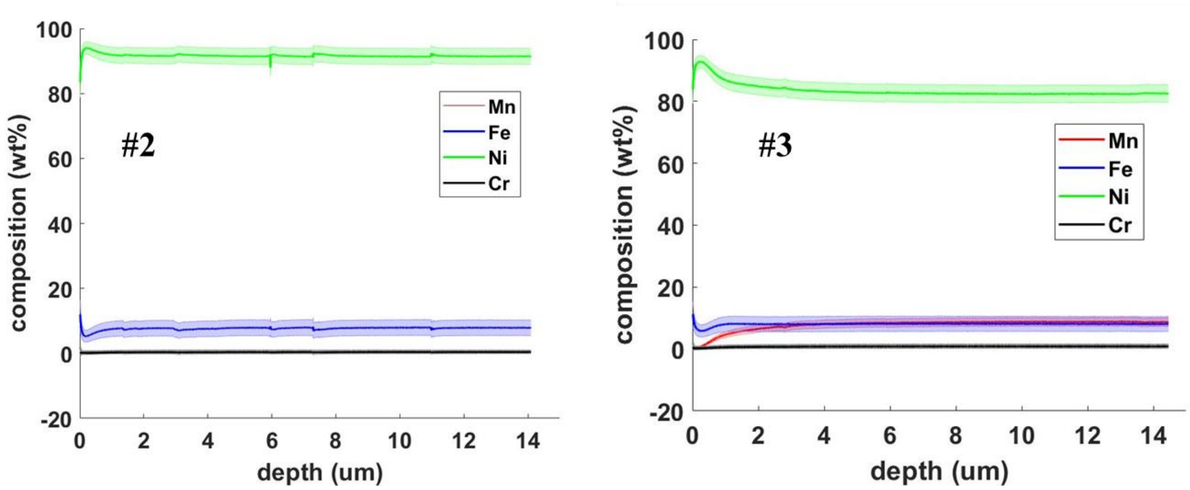

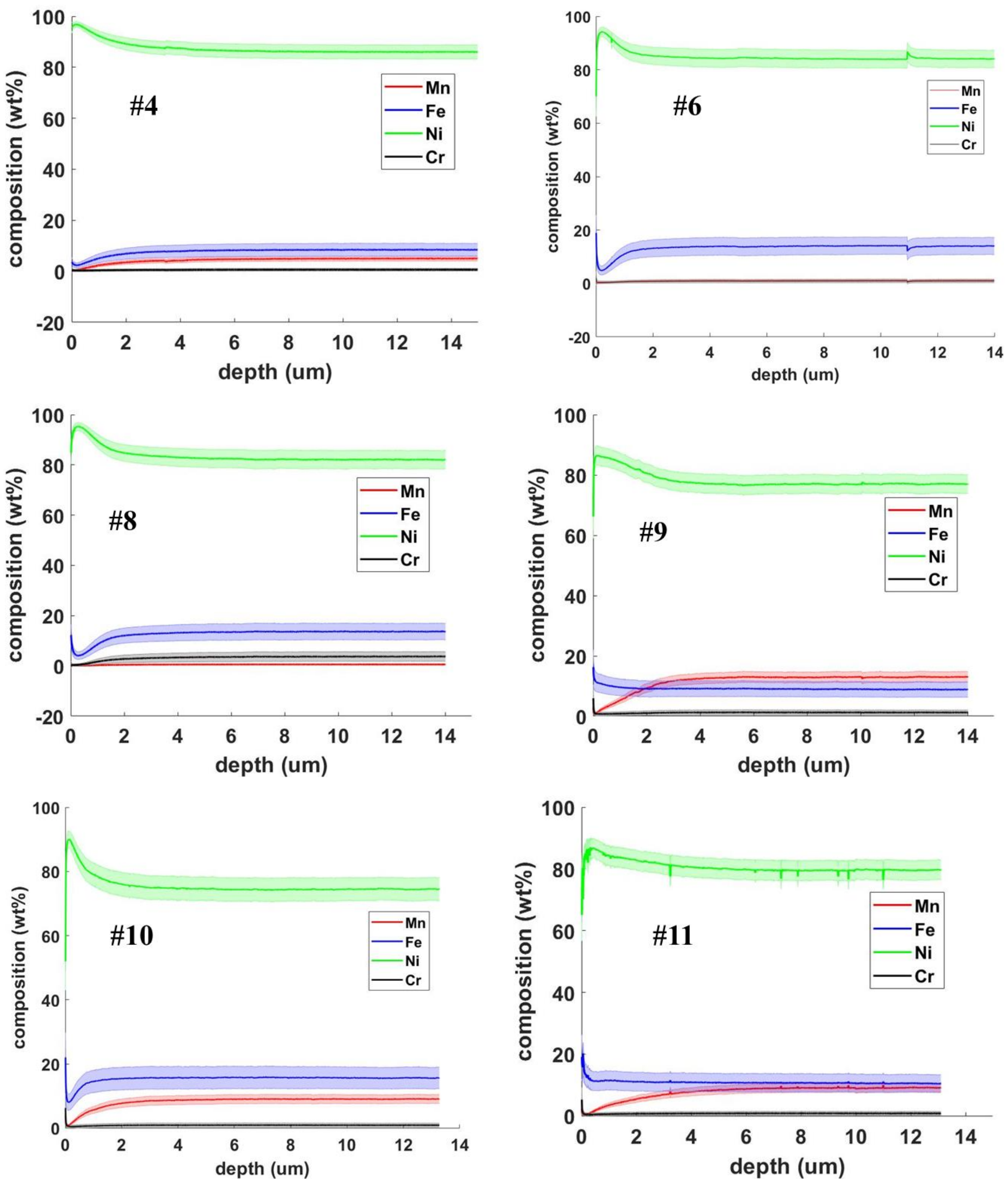
#4
#6
#8
#9
#10
#11
composition (wt%)
depth (um)
Mn
Fe
Ni
Cr

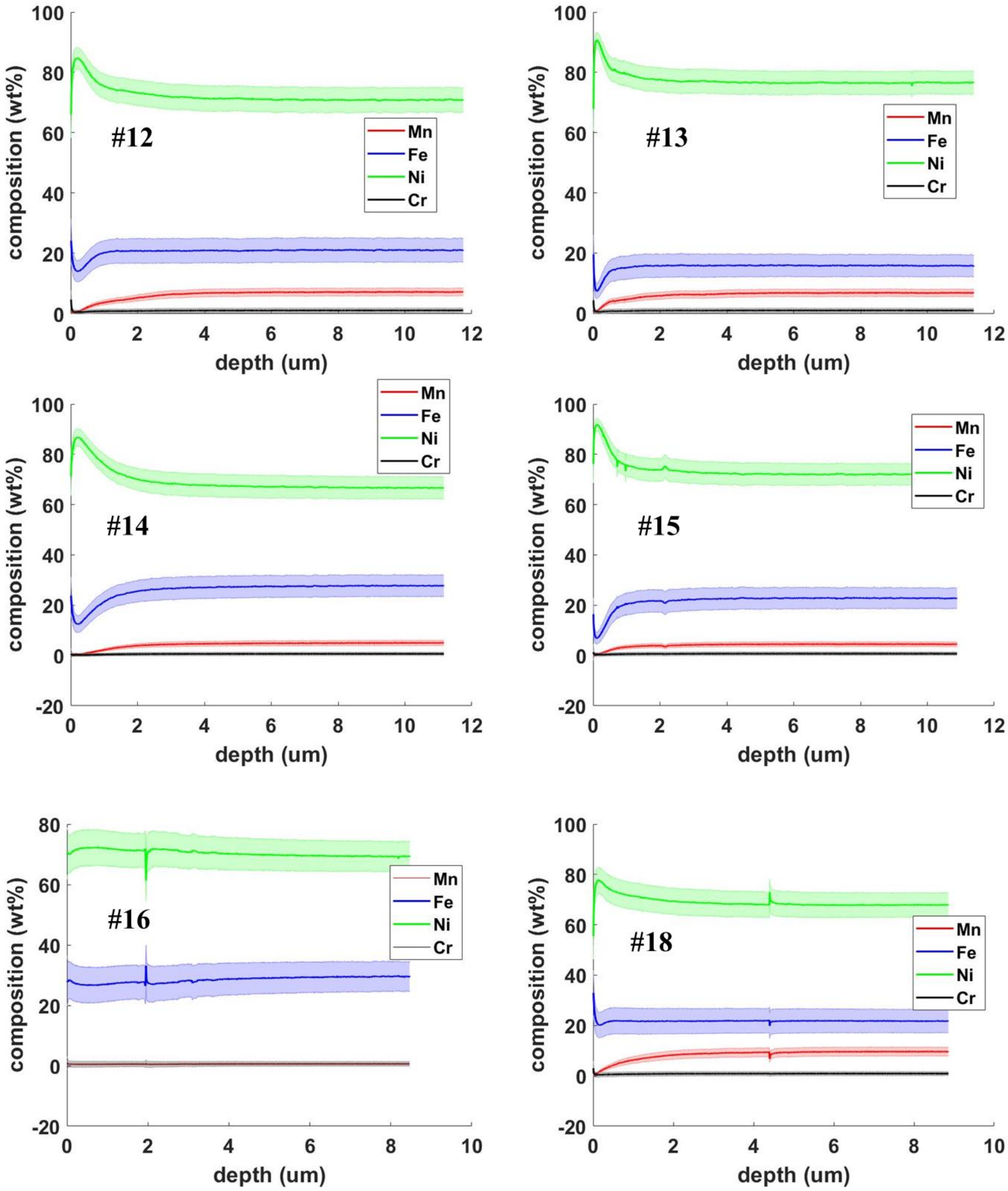
#12
#13
#14
#15
#16
#18
composition (wt%)
depth (um)
Mn
Fe
Ni
Cr
100
80
60
40
20
0
-20
0
2
4
6
8
10
12

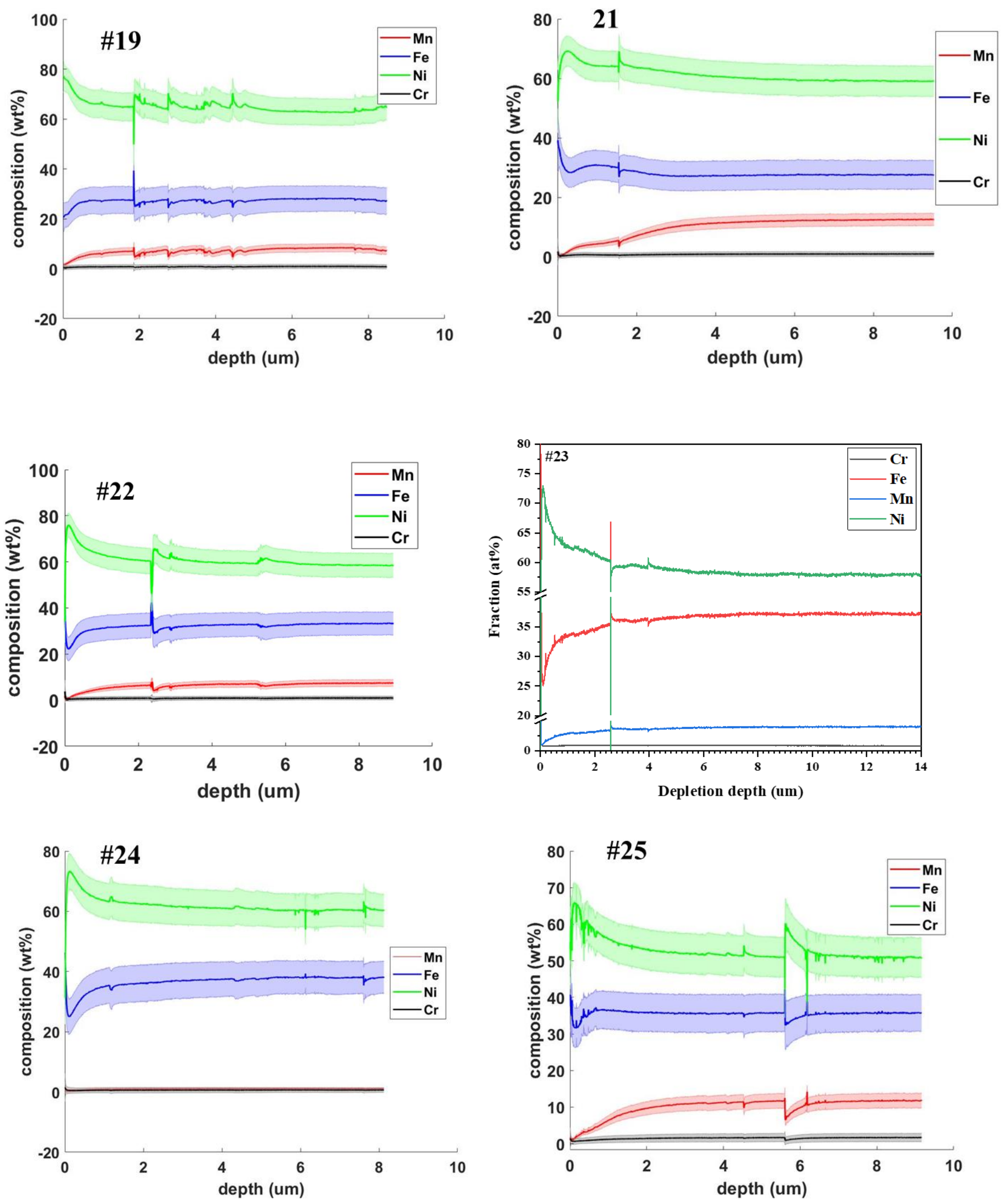

Figure S9. GDOES profiles of the post-corrosion printed alloys. The noises in the composition variation curves are induced by the air leakage during the GDOES analysis.

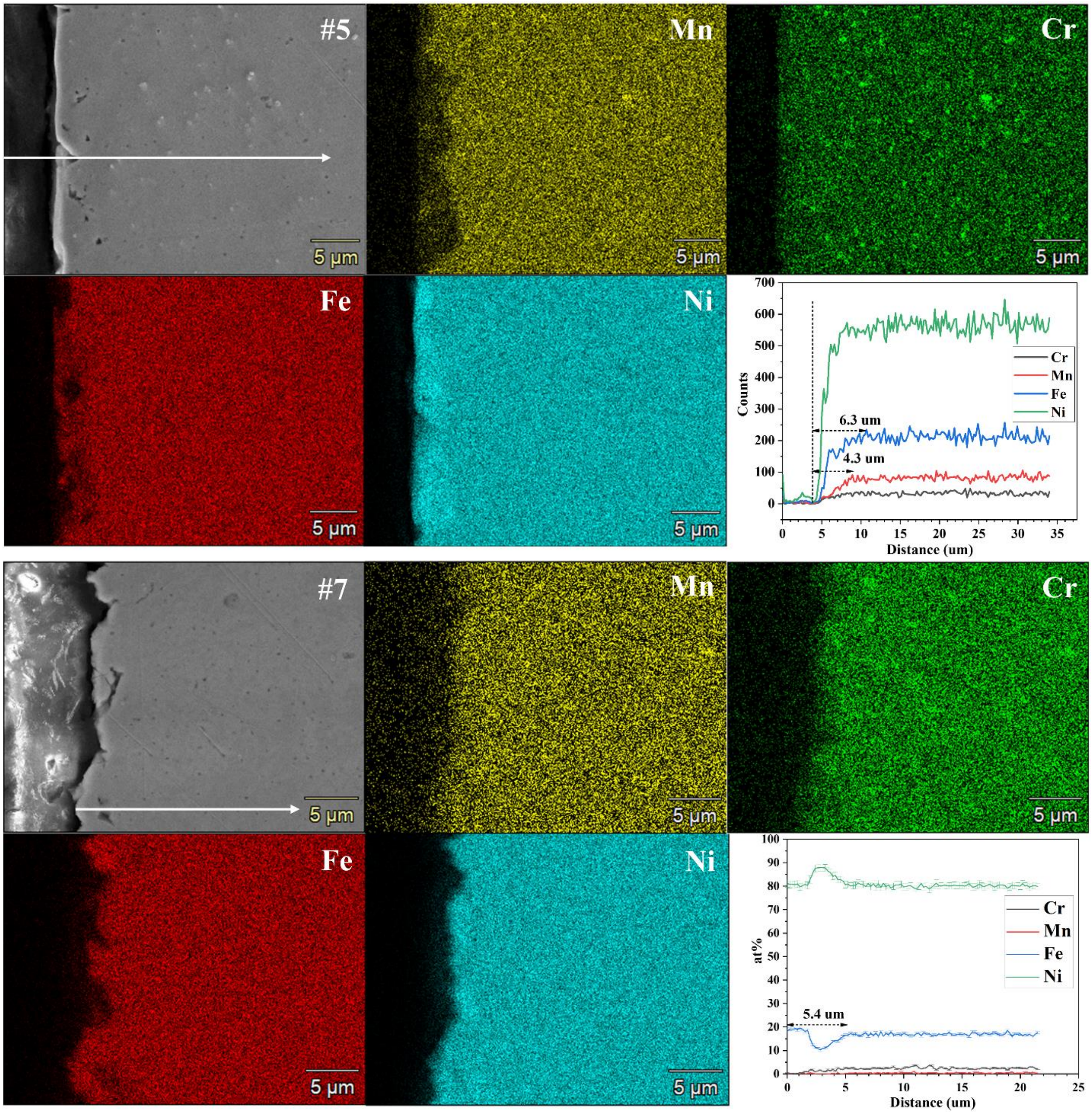
#5
Mn
Cr
Fe
Ni
5 μm
Counts
Distance (um)
Cr
Mn
Fe
Ni
6.3 um
4.3 um
#7
at%
5.4 um

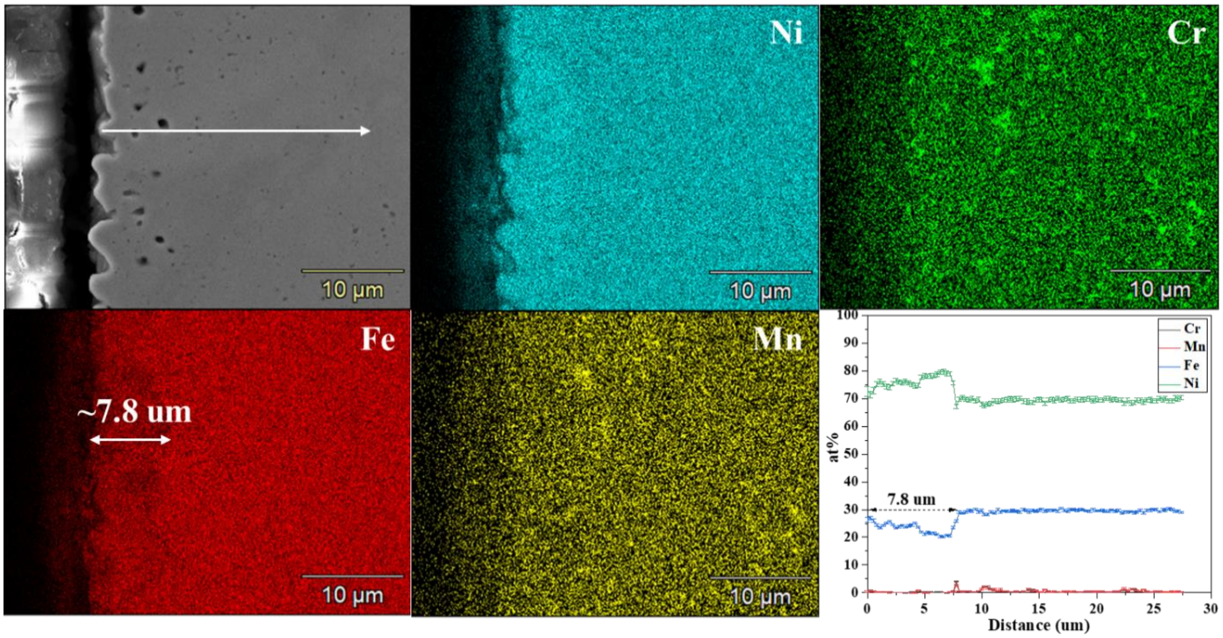

Figure S10. SEM, EDS compositional mapping, and EDS line scan of the near surface region of alloy #5, alloy #7, and alloy #17.

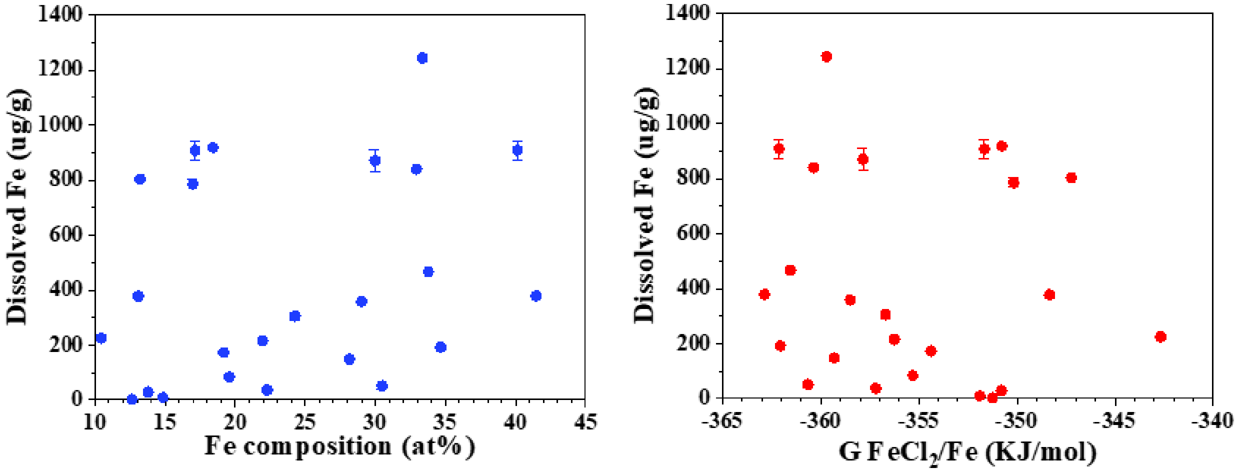

Figure S11. Correlations of Fe composition (in alloy) and G ($FeCl_2$/Fe) with the dissolution of Fe in molten salt.

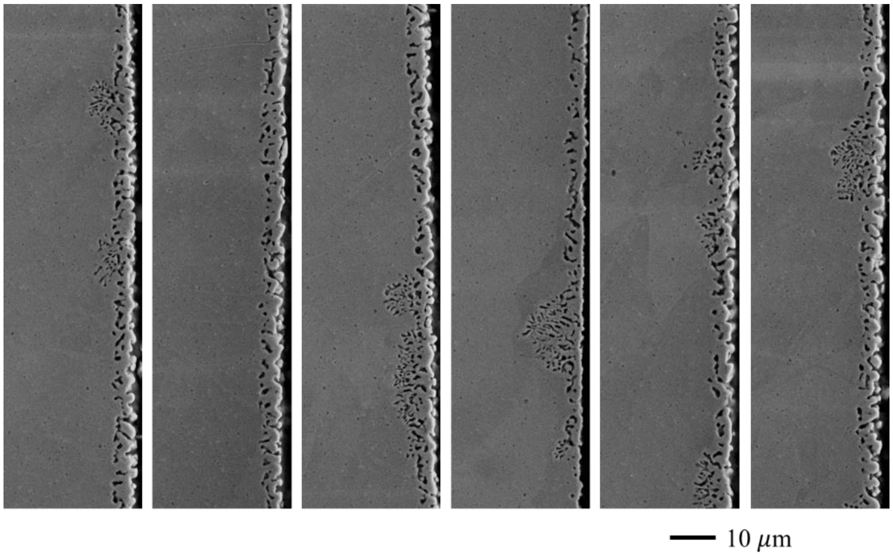

Figure S12. Corrosion of alloy#20 at different locations along its relatively large cross section area.
